# Influence of Vinyl Acetate Content and Melt Flow Index of Ethylene-Vinyl Acetate Copolymer on Physico-Mechanical and Physico-Chemical Properties of Highly Filled Biocomposites

**DOI:** 10.3390/polym15122639

**Published:** 2023-06-10

**Authors:** Pavel G. Shelenkov, Petr V. Pantyukhov, Matheus Poletto, Anatoly A. Popov

**Affiliations:** 1 Russian Academy of Sciences, Emanuel Institute of Biochemical Physics, Moscow 119334, Russia; shell1183@mail.ru (P.G.S.); anatoly.popov@mail.ru (A.A.P.); 2 Scientific laboratory “Advanced Composite Materials and Technologies”, Plekhanov Russian University of Economics, Moscow 117997, Russia; 3 Postgraduate Program in Engineering of Processes and Technologies (PGEPROTEC), University of Caxias do Sul (UCS), Caxias do Sul 95070-560, Brazil; mpolett1@ucs.br

**Keywords:** highly filled biocomposite, masterbatch, ethylene-vinyl acetate copolymer (EVA), wood flour, microcrystalline cellulose, water absorption, rheology, synthetic polymer, vegetable filler, mechanical properties

## Abstract

Highly filled biocomposites may be used as biodegradable masterbatches that manufacturers add to traditional polymers for making plastic goods more biodegradable. Biocomposites based on various trademarks of ethylene-vinyl acetate copolymer (EVA) and natural vegetable fillers (wood flour and microcrystalline cellulose) were studied. The EVA trademarks differed both in terms of the melt flow index and in the content of vinyl acetate groups. The composites were created as superconcentrates (or masterbatches) for the production of biodegradable materials based on vegetable fillers with polyolefin matrices. The filler content in biocomposites was 50, 60, 70 wt.%. The influence of the content of vinyl acetate in the copolymer and its melt flow index on the physico-mechanical and rheological properties of highly filled biocomposites was evaluated. As a result, one EVA trademark with high molecular weight that has a high content of VA was chosen because of its optimal parameters for creating highly filled composites with natural fillers.

## 1. Introduction

Products made of polymeric materials labeled as “biodegradable” appeared on the market several years ago. However, this labeling is not always true. Previous work examined the biodegradability of bags from supermarkets labeled as “biodegradable” [1]. Only bags made of biocomposite materials filled with starch have proven to be truly biodegradable. Biocomposites are promising materials that combine the advantages of synthetic polymeric materials and vegetable fillers [2]. The addition of a filler expands the scope of the material, reduces its cost and its impact on the environment [3]. Usually, a biodegradable polymer matrix is used to create biocomposites, to obtain a completely biodegradable biocomposite [3,4]. However, polyolefin matrices may also be used because they are significantly cheaper and usually outperform completely biodegradable polymer matrices in terms of consumer properties [5]. In this case, basic polymers, such as polyethylene [6], polypropylene [7,8], and copolymers of ethylene with octene [9] or vinyl acetate [10], may be used as a matrix. Copolymers have a number of advantages, for example, an ethylene-octene copolymer makes it possible to obtain composites filled with wood flour up to 70 wt.% [9,11]. It is known that ethylene-vinyl acetate copolymer (EVA) can be used as a compatibility agent to improve the adhesion of vegetable fillers to the polyethylene matrix [12,13,14]. For the industrial production of goods from such biocomposites, it is rational to use highly filled superconcentrates (or masterbatches) that the manufacturer of goods may add to pure polyethylene granules to obtain biocomposites with their required rheological and physico-mechanical parameters. Similarly, highly filled masterbatches with pigments and functional additives can also be obtained. With the maximum possible degree of filling in the matrix, it is important to ensure processing is completed on traditional equipment with acceptable physical and mechanical properties. When combining the maximum filling degree with the ability to process on traditional equipment, it is important to ensure that the finished product has acceptable physical and mechanical properties. Although masterbatches are not planned to be used in products in their pure form, their mechanical characteristics are very important because, after being diluted with a polymer, they will affect the properties of goods made from finished biocomposites. Weak adhesion of the filler to the polymer matrix in masterbatches, makes it impossible to obtain a biocomposite with acceptable mechanical characteristics, even with the addition of pure polyethylene. Therefore, it is important to strike a balance between the high degree of filling and the mechanical properties of the masterbatch.

To solve this problem, it is possible to vary the parameters of the polymer matrix and filler. Taking into account the previous positive results of using EVA as a compatibilizer, this research uses this copolymer. EVA trademarks differ in two parameters: melt flow index (MFI) and vinyl acetate (VA) content. Determining a method for controlling the properties of the masterbatch by adjusting these parameters using EVA is the main challenge of this study.

The choices among cellulose-containing fillers vary widely: flax shives, sunflower husks, tree leaves, oil flax stalks, coffee pucks, beet pulp, bagasse, bamboo, kenaf, cotton waste, and wood flour [3,4,15]. Taking into account that authors from different countries often use vegetable wastes from their own regions for the preparation of biocomposites, it was important to choose a filler that would be universal. For this reason, wood flour, as the most versatile and widely available vegetable filler, was used in this study. In addition to the main component–cellulose, wood flour also contains lignin, hemicellulose and a small amount of other components that stimulate biodegradation (proteins, fats). 

As a pure model filler with single-component composition, it is possible to choose between several polysaccharides: cellulose, starch, chitin, pectin, pullulan, alginate, or carrageenan [16]. Biocomposites containing starch and cellulose have been studied since 1974 [17]. Microcrystalline cellulose has a high bulk density and high heat resistance, which makes it easier to dose and process over a wide temperature range. According to a combination of these factors, microcrystalline cellulose was chosen as a model filler.

Manufacturers of plastic goods need a special additive that makes ordinary synthetic polymers more biodegradable. Adding cellulosic powders to melted polymers is technologically difficult,. and the addition of masterbatches in pellets to the pellets of pure polymers does not cause any technological problems. For this reason, manufacturers of single-use plastic products and packaging are in great need of highly filled biocomposites.

The published data were partially reported at the 5th Global Conference on Polymer and Composite Materials [18] and at the International Scientific Conference “FarEastCon-2019” [19]. 

## 2. Materials and Methods

For the preparation of highly filled biocomposites, five different trademarks of EVA produced by LG Chemical (South Korea) were used in this study. They differed from each other in MFI, as well as in VA content (Table 1). The trademarks were collected by the same manufacturer to investigate the role of MFI in EVA trademarks 28150, 28025, and 28005, or the role of VA content comparing 28150 versus 19150 or 28005 versus 15006. 

Wood flour (WF), a mixture of deciduous trees (mainly birch), was used as a filler for superconcentrates. Wood flour was supplied by LLC Novotop (Moscow, Russia). Microcrystalline cellulose (MCC) produced by LLC Progress (Kemerovo, Russia) under the trademark MCC-101, was used as a pure model filler, due to its single-component composition. Both fillers were preliminarily dried for 3 h in an oven at 105 °C and sifted through a sieve with a mesh size of 100 μm. The chemical composition of the fillers is presented in Table 2.

**Figure 1:**
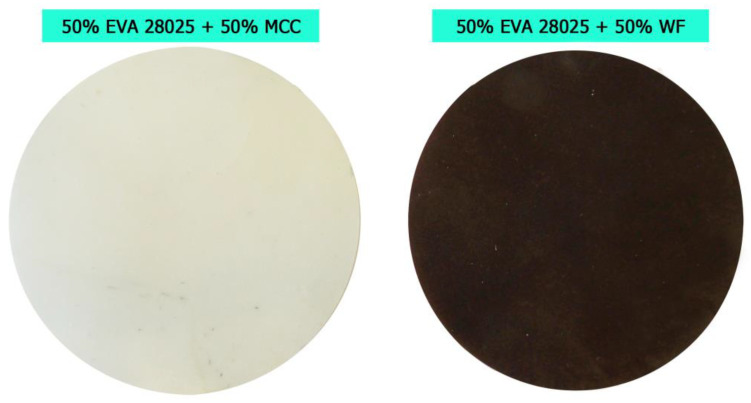
Samples of superconcentrates with microcrystalline cellulose (**left**) and wood flour (**right**).

Three highly filled biocomposites, differing in the content of their filler (50, 60 and 70% weight) were prepared with each of the five EVA grades. Biocomposites were prepared at heated mixing rolls UBL6175BL (China). The temperature on the rollers was 130 and 150 °C, respectively, and the rotation speed was 8 rpm. After cooling, the resulting composites were milled using the knife mill Vibrotechnik RM-120 (Russia). Next, the material was compressed in a thermohydraulic press GOTECH GT-7014-H30C (Taiwan) at a temperature of 140 °C and a load of 40 kgf/cm^2^ for 1 min. This process resulted in flat white sheets for cellulose biocomposites and dark brown sheets for wood flour biocomposites (Figure 1). The thickness of the obtained sheets varied from 0.4 to 0.5 mm. Samples were stamped and cut out of the resulting sheets for this research.

Micrographs of the powders were obtained via scanning electron microscopy (SEM) to determine the shape of the particles. Tescan Vega 3SB (Czech Republic), a fully computer-controlled scanning electron microscope with a traditional tungsten thermal cathode, designed for research in high vacuum, was used for this research. To study dispersed materials, the samples were fixed on the microscope stage. Fixation of the materials involved the application of double-sided carbon conductive tape. One side of the adhesive tape was glued to the object stage of the electron microscope, and the sample was adhered on a thin layer on its reverse side. The following parameters were used: accelerating voltage–20 kV; current intensity–low current; working distance–15 mm.

The distribution of filler particles in EVA matrices was studied using a Carl Zeiss Axio Imager Z2M optical microscope with AxioVision (Germany), with a magnification of 300× in transmitted light.

A Fritsch Analysette 22 laser instrument (Germany) was employed to determine the particle size and plot the distribution curve in accordance with ISO 13320 Particle Size Analysis-Laser Diffraction Methods.

Cellulose-containing fillers are heated up to 150 °C during the polymer processing, therefore, it is important to study the temperature of their thermal degradation. Thermogravimetric analysis (TGA) was carried out to study the thermal stability of the fillers. The studies were carried out on a Netzsh TG 209 F1 Iris synchronous thermal analyzer (Germany). The weighed sample was heated from 25 °C to 950 °C at a rate of 20 °C/min. First, a dry nitrogen purge was performed. Upon reaching a temperature of 850 °C, a transition from dry nitrogen to “synthetic air” occurred. When the air passed, the existing soot was burned. As a result, the mass of the remaining substance was recorded.

The specific gravity (density) of the fillers was determined by pycnometric weighing in n-hexane. The determination method described in ISO 2811-1:2011 was taken as the basis. An empty 25 mL pycnometer was weighed using analytical balance AND GH-252 (Japan). Subsequently, filler powder was poured into the pycnometer (1/3 of the volume of the pycnometer) and the pycnometer with filler [m (pyc+fil)] was weighed. Next, the pycnometer was filled with n-hexane up to the 25 mL mark and weighed again [m(pyc+fil+hex)]. The completely filled pycnometer with n-hexane and without filler [m(pyc+hex)] was also weighed. The density of the filler powder was calculated by the formula:

$$\rho_{\mathrm{fil}}=\frac{m(fil)}{\left[m\left(pyc+hex\right)+m\left(fil\right)\right]-m(pyc+fil+hex)}\cdot \rho_{\mathrm{hex}}$$

The density of the filled composites was determined via hydrostatic weighing in ethanol according to ISO 1183-1:2019. To determine the density, analytical balance AND GH-252 (Japan) was used. The weight of the sample was determined in air and in ethanol, and the density of biocomposite was determined using the following formula:

$$\rho_{\mathrm{biocomposite}}=\frac{m(biocomposite\;in\;air)}{m\left(biocomposite\;in\;air\right)-m\left(biocomposite\;in\;ethanol\right)}\cdot \rho_{\mathrm{ethanol}}$$

The presence of voids in biocomposites was determined by the difference in densities. The experimentally measured density of biocomposite was subtracted from the calculated density of biocomposite. The calculated density was defined as the sum of the densities of all components multiplied by coefficients equal to their percentage in the composition. The difference indicated the presence of voids in biocomposite.

∆ρ = ρ(calculated) − ρ(experimentally measured)

Water absorption is an indirect characteristic of biodegradation. Biodegradation is a complex process that includes the penetration of water into the bulk of biodegradable material. Water absorption was measured according to ISO 62:2008 “Plastics-Determination of water absorption”. Before testing, the samples were dried for 24 h in an oven at a temperature of 50 °C. Next, their masses were determined and the samples were placed in distilled water at room temperature (23 ± 2 °C). Every 5 days for a month, the samples were removed from distilled water and blotted with filter paper to remove water drops on the surface of the sample. Next, the samples were weighed. The water absorption of the samples was determined in mass % according to the difference in masses.

The rheology of biocomposites was assessed via melt flow index (MFI) using a Metrotex MT 091 capillary viscometer (Russia). The MFI was determined according to ISO 1133-1:2011, where *T* = 190 °C, *F* = 2.16 kg and 10.0 kg, and *d* = 2.095 mm. The MFI depends on the viscosity of the polymer at a given temperature, and the viscosity directly depends on the molecular weight η = K∙M, where K is the constant of proportionality and M is the molar mass [22].

The mechanical properties of the biocomposites were determined using a GOTECH AI-7000M universal testing machine (Taiwan). The tensile strength and elongation at break were determined, and crosshead velocity was 100 mm/min. The test specimens were type 5A according to ISO 527-1:2012 and the dimensions of the samples were: 75 mm × 4 mm × 0.4 mm. The data for seven samples were averaged.

All laboratory tests were carried out using scientific equipment at the Center of Shared Usage ⟪New Materials and Technologies⟫ at Emanuel Institute of Biochemical Physics and Joint Research Center at Plekhanov Russian University of Economics.

## 3. Results and Discussion

**Figure 2:**
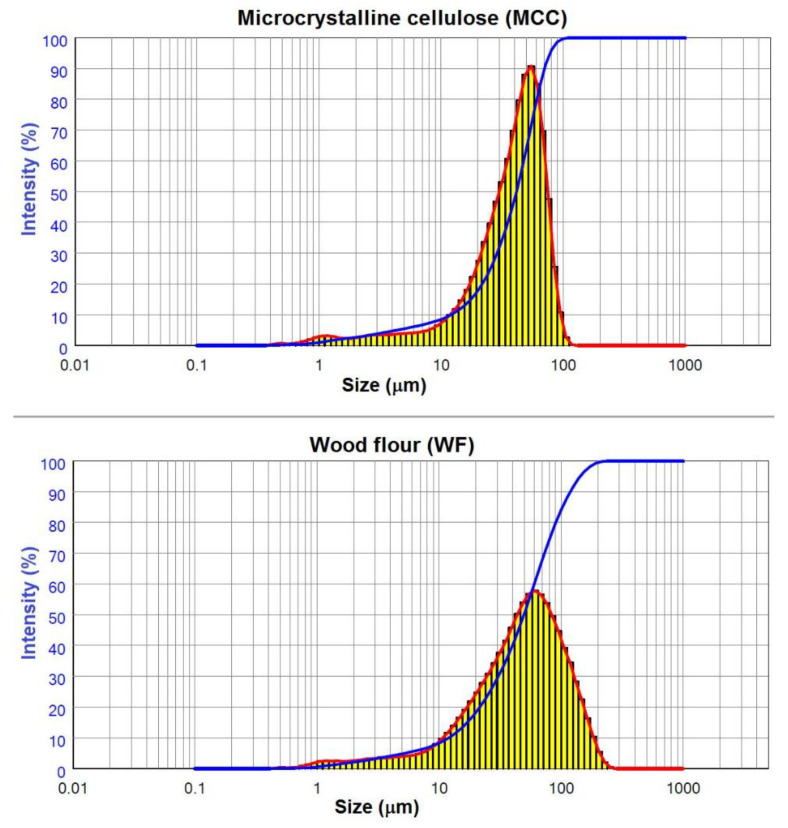
Particle size distribution curves.

Despite the fact that the particles of both fillers were sifted through the same sieve with a mesh diameter of 100 µm, their size distribution was unequal (Figure 2). MCC had a narrow distribution of particles, while WF had a wider distribution. The proportion of fine particles smaller than 20 µm is the same for both fillers at 20%. However, the proportion of coarse fraction 75–100 microns differed significantly: 6% for MCC versus 13% for WF. Furthermore, no particles larger than 100 µm were found in MCC, and 16% of such particles were found in WF. This can be explained by the elongated shape of the WF particles, which are able to pass through the sieve cell in diameter, while their length exceeds the cell diameter. Thus, WF had larger particles than MCC; therefore, volume moment mean D [3,4] for MCC was 41.6 µm, and 58.3 µm for WF. 

**Figure 3:**
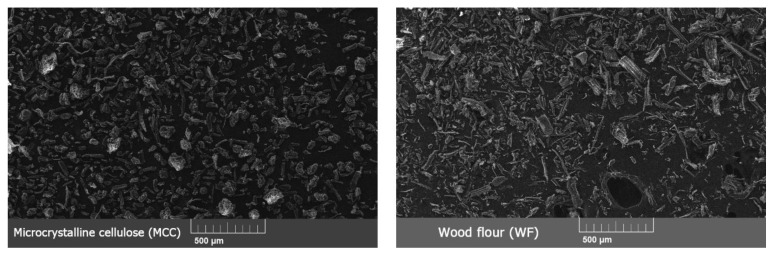
Microphotographs of particles of microcrystalline cellulose (MCC) and wood flour (WF) made via scanning electron microscopy (SEM).

The shape of the fillers’ particles also differed–MCC had both cylindrical and close-to-spherical shape particles with a smooth surface, while WF particles had an uneven cylindrical shape with rough surface (Figure 3). Smooth MCC particles should have fewer voids in ready-made biocomposites than rough and branched WF particles. On microphotographs with the same magnification, it is visually apparent that, in general, WF particles are slightly larger than MCC particles, which confirms the results of the distribution of particles on a laser analyzer.

**Figure 4:**
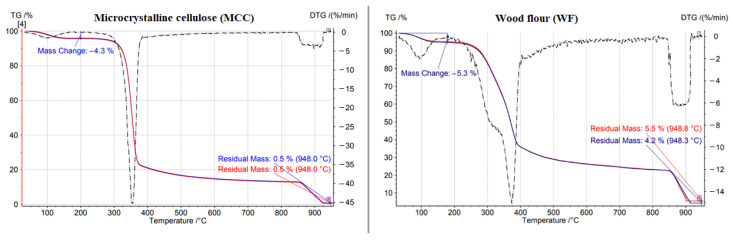
TGA thermograms of microcrystalline cellulose and wood flour.

A study of the thermal stability of the fillers by the thermogravimetric analysis (TGA) was performed; the thermograms of the MCC and WF are presented in Figure 4. Despite the fact that the samples were dried in an oven before the study, they still contained 4–5% moisture, which starts evaporating from 70 °C. The start of thermal degradation in WF occurs at a lower temperature (270 °C) than that of MCC (328 °C). This can be explained by the presence of lignin and pentosans in WF. Pentosans are the least resistant to heat and decompose first, followed by hemicellulose, and finally, lignin [20]. Thermal degradation of hemicellulose occurs in the range of 270–325 °C, while the same occurs for cellulose in the range of 305–375 °C, and lignin in the range of 270–500 °C. Due to the degradation of three main components in the range of 270–375 °C, a wide shoulder was found in thermogram. At 500 °C organic matter decomposes to soot. The sharp increase after 850 °C is explained by the switching of the medium from nitrogen to synthetic air. In the presence of oxygen, soot was turned to inorganic ash. The residual mass of WF is greater (4.2–5.5 wt.%) than that of MCC (0.5 wt.%). Various inorganic substances are present in wood flour, but their total content is lower than 5%. Most likely, these are calcium, potassium, sodium, and magnesium; however, a detailed elemental analysis of the ash residue was not carried out in this study.

**Figure 5:**
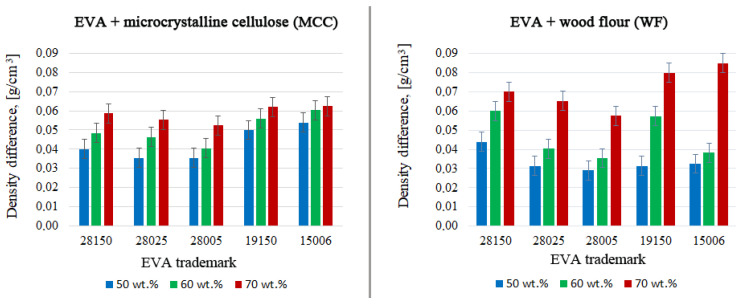
The defectiveness of biocomposites.

The final characteristic determined in pure fillers was the specific gravity (density). The density of WF was 1.44 g/cm^3^ and the density of MCC was 1.50 g/cm^3^. The calculated density of biocomposites was determined based on the densities of copolymers and fillers. The defectiveness of biocomposites was determined as the difference between the calculated and experimentally obtained density. Density defectiveness expresses the amount of cracks and voids encapsulated in the samples. Figure 5 presents the defectiveness of all prepared biocomposites. The defectiveness of biocomposites increases with an increase in the filler content in each series of samples. The defectiveness of samples filled with WF and MCC were at the same level, except for those with 70 wt.% of WF. The composite with 70 wt.% WF was extremely defective in all matrices. It can be concluded that for the preparation of homogeneous biocomposites, the maximum degree of filling with WF is 60 wt.%, while MCC can be filled up to 70 wt.% and possibly higher. 

The defectiveness of both biocomposites slightly increased with an increase in the MFI of EVA, with the same VA content. Comparing biocomposites with the same MFI, but different VA content (28150 vs. 19150 and 28005 vs. 15006) in EVA, it can be seen that in biocomposites with MCC, a decrease in VA content increases the defectiveness. This is explained by chemical binding of VA groups with cellulose. However, this trend was not observed in biocomposites with WF. The content of cellulose in WF (37–56%) is lower than in MCC (100%). Cellulose fibers in WF are isolated from the external environment by lignin. That is why VA groups from polymers could not bind to the cellulose of WF.

**Figure 6:**
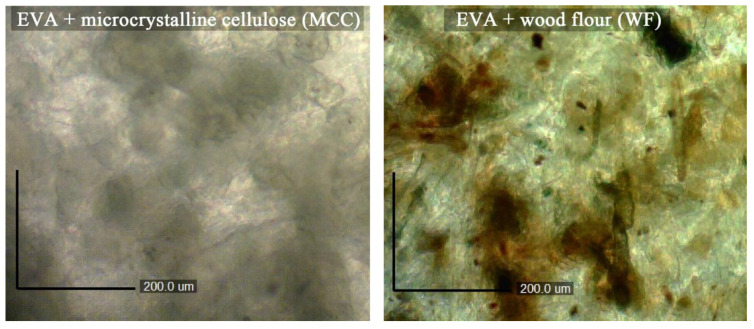
Microphotographs of biocomposites in transmission light (300×).

Particles of WF and MCC were distributed differently in the polymer matrix. This can be seen in the micrographs of the films. Figure 6 presents micrographs of pressed films (thickness 100 μm) of biocomposites based on EVA 28025 filled with MCC and WF. Biocomposites based on other EVA trademarks had the same particle distribution in polymer matrices. The structure of biocomposites with MCC are homogeneous. At the same time, dark spots of various geometric shapes are visible in the micrograph of WF. These are agglomerates of wood flour particles, which form a heterogeneity in the structure. WF particles strongly adhered to each other because of the branched and rough surface of the particles (Figure 3), forming large agglomerates. Such agglomerates cause the formation of imperfections at high rates of filling and make it impossible to obtain composites with a filling of more than 60 wt.%. Due to agglomerates, an uneven structure is formed in the sample, which reduces both the technological (MFI) and mechanical (elongation at break) properties. 

A similar problem was discovered in earlier research. In Zykova et al [23], WF was sifted through a sieve with a mesh size of 200 µm, 140 µm, and 80 µm. Subsequently, biocomposites were prepared with a complex filler fraction of 0–200 μm and individual fractions of 0–80, 80–140, and 140–200 μm, respectively. Biocomposites made from the complex fraction were defective due to unseparated particles in the form of agglomerates. In this study, a finer fraction (0–100 µm) was used, but nonetheless, there are agglomerates. Most likely, further screening through 80, 60 and 40 µm sieves, respectively, would help to solve this problem, even if all the parts were mixed together after fractionation. Such work will be carried out in the future. 

**Figure 7:**
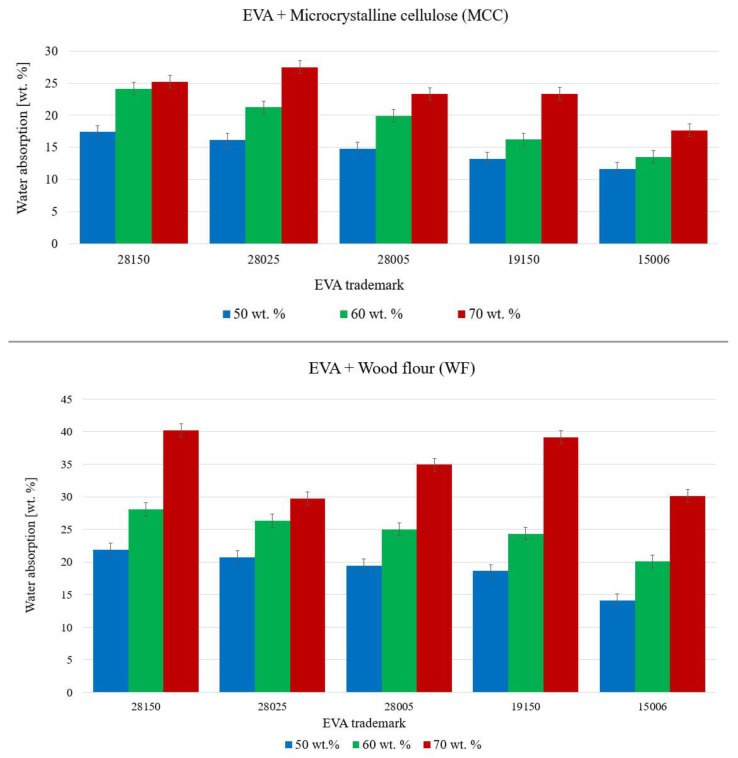
Water absorption of biocomposites after days of exposure in water.

The water absorption index makes it possible to study the structure of biocomposites (Figure 7). Water penetrates into the pores of the fillers, as well as cracks and voids in the polymer matrix and the interface. The water absorption of biocomposites increased both with an increase in the filler content and with an increase in the content of VA in the initial EVA. This is noticeable when comparing the water absorption of two pairs of biocomposites with the same MFI (28150 vs. 19150 and 28005 vs. 15006). The polar nature of VA allows biocomposites to absorb more moisture at a higher VA content. Furthermore, a regular increase in water absorption with an increase of MFI was found. Comparing the fillers, it was noted that the water absorption of biocomposites with WF was almost 1.5 times higher than that of biocomposites with MCC. This is due to the more porous structure of WF particles, and thus the water absorption of WF was higher than that of MCC both in its pure form and in biocomposites. Moisture penetrates into the microcavities inside the biocomposite, so the most defective biocomposites with 70% WF showed the highest percentage of water absorption. This indicates a potentially high rate of biodegradation under high moisture environmental conditions, however, due to their low mechanical properties, they are unlikely to be used to produce commercial goods.

The melt rheology was evaluated by the changes in MFI; 50% filled biocomposites flowed at a standard load of 2.16 kg, however, at a higher filler content (60 and 70%), they stopped flowing. For 60 and 70% filled biocomposites, the load was increased to 10 kg. For the comparison of 50% to 60 and 70% biocomposites, the MFI of 50% biocomposites with a load of 10 kg were also obtained. The MFI of highly filled biocomposites are shown in Table 3. For 50% biocomposites the values obtained at two different loads (10.0 and 2.16 kg), are presented. When 50% of MCC was added to the EVA, the MFI of the composite dropped by approximately one order of magnitude, and when 50% WF was added, the MFI dropped by two orders of magnitude. For biocomposites MCC_28025 and MCC_28150 it can be seen that an additional 10% of the filler leads to a decrease in the MFI by 30–50 times. The difference in the flow of biocomposites with different fillers is explained by the shape of the filler particles. WF particles had an L/D (length to diameter) ratio of approximately 2.5 [23], larger than that of spherical MCC particles (L/D ≈ 1). Spherical particles flow much better in a liquid medium than elongated particles, which can block the flow in a capillary. It is obvious that an increase in filler content leads to a noticeable decrease in the MFI. The absence of a flow for 70% biocomposites indicates the impossibility of obtaining finished goods from these materials using traditional methods (extrusion, injection molding). Therefore, biocomposites with 70% filler are proposed to be used only as an easily introduced additive for the other polyolefins.

**Figure 8:**
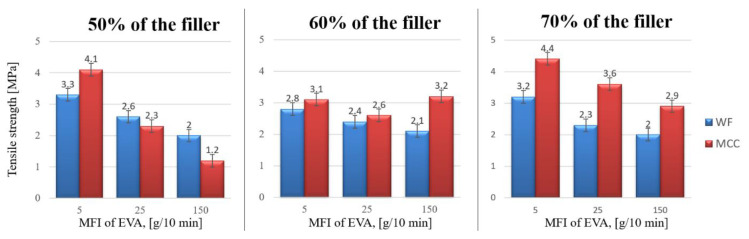
The relationship between the tensile strength of biocomposites and MFI of EVA.

Mechanical characteristics of the obtained biocomposites were studied in order to assess the adhesion of the filler to the polymer matrix, and the homogeneity of the system. With the same content of VA in EVA, the tensile strength of biocomposites increased as the MFI of the initial EVA trademark decreased i.e., its molecular weight increased (Figure 8). This is consistent with the strength of initial copolymers–an increase in MFI leads to a decrease in tensile strength; basic characteristics of initial copolymers are presented in Table 1. The stress-strain curves were provided in a supplemental file. 

**Figure 9:**
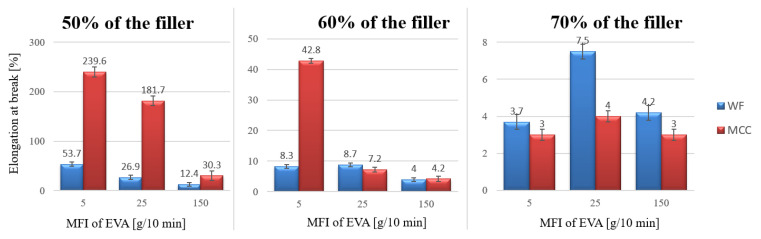
The relationship between the elongation at break of biocomposites and MFI of EVA.

The influence of the filler content on the strength of biocomposites has not been established. Fibrous filler particles should strengthen the polymer, while irregularly shaped particles, on the contrary, reduce the strength of the polymer due to its inability to withstand the load transmitted by the polymer matrix [24]. For some 70% biocomposites the strength was rather high, which can be explained by the formation of a continuous phase consisting of fillers’ particles. The filler content strongly affects the elongation at break: the more filler, the lower the elongation (Figure 9). Elongation at break dropped by one order of magnitude for both fillers with an increase of filler content from 50 wt.% to 60 wt.%. These results are consistent with previous work, where an increase in the filler concentration from 30 to 70 wt.% also decreased the elongation at break dramatically [9]. The same regularity was observed in biocomposites with cotton fiber: with increasing filler concentration, the elongation at break decreased [25]. Usually, a decrease in elongation is explained by a decrease in the deformability of the interface between the polymer and filler phases [24]. The filler content had more influence on the elongation for biocomposites with MCC than for those with WF. WF particles had a much larger L/D ratio than MCC, resulting in a less uniform distribution of the filler in the matrix and more defect formations. When the samples are elongated, the macromolecules straighten out in the free space, and the solid particles of the filler do not allow them to do this. Smooth and spherical MCC particles hinder the straightening of macromolecules less than branched rough WF particles. For initial EVA copolymers, as the MFI decreases, the elongation at break also decreases (compare trademarks 28,005, 28,025, 28,150 in Table 1). For biocomposites, on the contrary, the higher the MFI of the initial EVA trademark, the lower the elongation at break. The molten polymer penetrates into the pores of the fillers during mixing at heating rolls, and the part of the polymer that did not penetrate into the filler forms its own phase. Fluid trademarks fill the pores of the filler well, therefore, at high filler content, where there is no free polymer left to form its own phase. More porous and loose WF particles adsorb much more polymer than smooth MCC particles. Therefore, in biocomposites with WF, there remains less free polymer capable of forming its own phase. This assumption explains the higher elongation at break of MCC compared to WF. For biocomposites with 70% filler, these regularities do not always hold true because their very defective structure allows for possible fluctuations (Figure 5).

**Figure 10:**
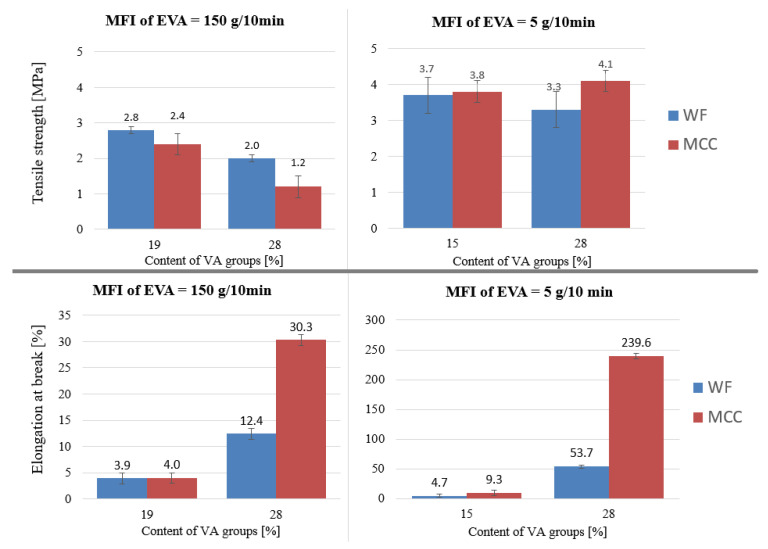
The relationship between the tensile strength or elongation at break of biocomposites and the content of VA in EVA.

Changes in the tensile strength and elongation at break of biocomposites with a change in the content of VA in EVA were evaluated under the condition that the initial MFI was constant (Figure 10). With an increase in the content of VA in EVA, the elongation of biocomposites increased significantly. Considering that the elongation at break for the initial copolymers was the same (800% for 15,006 and 28,005), it is apparent that VA groups chemically bonded with filler particles, increasing their adhesion with the matrix and improving the uniformity of their distribution over the matrix. Biocomposites based on EVA trademarks with a higher VA content have a higher elongation at break. The strength of biocomposites slightly decreases with an increase of VA, which can be explained by the tensile strength of the initial EVA trademarks: 4 MPa for 28,150 versus 7 MPa for 19,150 (Table 1).

## 4. Conclusions

Highly filled biocomposites have the potential to be used as additives to pure polymers. The manufacturers of plastic goods may add them to ordinary plastics at the final stage, thereby customizing goods and making them slightly more biodegradable. 

The most effective EVA trademarks for preparing highly filled biocomposites with natural fillers are high molecular weight trademarks that have a high VA content at a low MFI. Biocomposites, based on EVA trademarks with low VA content and high MFI have a great number of defects; these data are confirmed by the indicators of water absorption and tensile strength of biocomposites. With the same VA content, the tensile strength of biocomposites increases with a decrease in the MFI of the initial EVA trademark, while the water absorption decreases. The elasticity of biocomposites increases with a decrease in the MFI of the initial EVA trademark. The most acceptable rheological properties of highly filled biocomposites are at 50 wt. % of filling; biocomposites with higher filler content are difficult to process via common methods (injection molding, extrusion). Biocomposites with MCC show better deformation-strength and rheological characteristics than those with WF, and furthermore, MCC has a higher decomposition temperature. At the same time, biocomposites based on MCC have lower water absorption than WF based. The lower the water absorption of biocomposites, the lower their biodegradability.

Among all the studied trademarks of EVA, EA 28025 is optimal for preparing highly filled biocomposites. Such biocomposites have an optimal balance of sufficient mechanical properties, high water absorption, and MFI, which makes it possible to process them via common methods.

## Figures and Tables

**Table 1:** Characteristics of EVA trademarks.

Characteristics	EVA Trademarks				
	EA28150	EA28025	ES28005	EA19150	EC15006
Content of VA, [%]	28	28	28	19	15
MFI [g/10 min]	150	25	5	150	6
Tensile strength, [MPa]	4	9.5	13.5	7	16
Elongation at break, [%]	900	850	800	800	800
Density [g/cm^3^]	0.946	0.951	0.951	0.940	0.936
Melting temperature [°C]	70	69	72	80	89

**Table 2:** Chemical composition of the fillers.

Fillers	Cellulose [%]	Lignin [%]	Hemicellulose [%]	Protein [%]	Fats [%]	Reference
Wood flour (WF)	37–56	18–22	25–40	0.3	0.4	[20,21]
Microcrystalline cellulose (MCC)	100	-	-	-	-	-

**Table 3:** Melt flow index of highly filled biocomposites (Load = 10 kg, T = 190 °C).

EVA Content [wt. %]	Melt Flow Index (MFI) of EVA [g/10 min]	Melt Flow Index (MFI) of Biocomposites [g/10min]					
		50 wt. %		60 wt. %		70 wt. %	
		MCC	WF	MCC	WF	MCC	WF
28	5	8.7/ 0.5 *	2.9/ 0.08 *	2.5	0.3	No flow	No flow
28	25	21.3/ 4.0 *	7.1/ 0.28 *	10.0	0.8	0.19	No flow
28	150	95.0/ 17.1 *	18.1/ 0.65 *	25.0	0.9	0.76	No flow
15	6	10.6/ 1.5 *	3.4/ 0.03 *	3.6	0.3	No flow	No flow
19	150	92.0/ 16.7 *	16.5/ 0.15 *	22.0	0.8	0.43	No flow

* Load = 2.16 kg, T = 190 °C.

## Data Availability

Data sharing not applicable.

## Supplementary Materials

The following are available online at https://www.mdpi.com/article/10.3390/polym15122639/s1.

## References

[1] Mastalygina E., Abushakhmanova Z., Poletto M., Pantyukhov P. (2023). Biodegradation in Soil of Commercial Plastic Bags Labelled as “Biodegradable”. Mater. Res..

[2] Mohanty A.K., Misra M., Drzal L.T., Mohanty A.K., Misra M., Drzal L.T. (2005). Natural Fibers, Biopolymers, and Biocomposites.

[3] Brebu M. (2020). Environmental Degradation of Plastic Composites with Natural Fillers—A Review. Polymers.

[4] Faruk O., Bledzki A.K., Fink H.-P., Sain M. (2014). Progress Report on Natural Fiber Reinforced Composites. Macromol. Mater. Eng..

[5] Popov A.A. (2021). Biodegradable Polymer Compositions Based on Polyolefins. Polym. Sci. Ser. A.

[6] Hejna A., Korol J., Kosmela P., Kuzmin A., Piasecki A., Kulawik A., Chmielnicki B. (2021). By-Products from Food Industry as a Promising Alternative for the Conventional Fillers for Wood–Polymer Composites. Polymers.

[7] Schneider M., Finimundi N., Podzorova M., Pantyukhov P., Poletto M. (2021). Assessment of Morphological, Physical, Thermal, and Thermal Conductivity Properties of Polypropylene/Lignosulfonate Blends. Materials.

[8] Mastalygina E.E., Popov A.A. (2017). Mechanical Properties and Stress-Strain Behaviour of Binary and Ternary Composites Based on Polyolefins and Vegetable Fillers. Solid State Phenom..

[9] Zykova A., Pantyukhov P., Popov A. (2017). Ethylene-Octene Copolymer-Wood Flour/Oil Flax Straw Biocomposites: Effect of Filler Type and Content on Mechanical Properties. Polym. Eng. Sci..

[10] Zimmermann M.V.G., Turella T., Santana R.M.C., Zattera A.J. (2014). Comparative Study between Poly(Ethylene-Co-Vinyl Acetate)—EVA Expanded Composites Filled with Banana Fiber and Wood Flour. Mater. Res..

[11] Zykova A., Pantyukhov P., Popov A. (2016). Mechanical Properties of Ethylene-Octene Copolymer (EOC)—Lignocellulosic Fillers Biocomposites in Dependence to Filler Content. AIP Conf. Proc..

[12] Li D., Li J., Hu X., Li L. (2012). Effects of Ethylene Vinyl Acetate Content on Physical and Mechanical Properties of Wood-Plastic Composites. Bioresources.

[13] Pantyukhov P.V., Monakhova T.V., Kolesnikova N.N., Popov A.A., Nikolaeva S.G. (2013). Destruction of Composite Materials Made of LDPE and Lignocellulosic Fillers. J. Balk. Tribol. Assoc..

[14] Shelenkov P.G., Pantyukhov P.V., Popov A.A. (2021). Mechanical Properties of Bio-Composites Based on Polymer Blends of Ethylene-Vinyl Acetate Copolymer and Polyethylene with Natural Fillers. Solid State Phenom..

[15] Faruk O., Bledzki A.K., Fink H.-P., Sain M. (2012). Biocomposites Reinforced with Natural Fibers: 2000–2010. Prog. Polym. Sci..

[16] Aleksanyan K.V. (2023). Polysaccharides for Biodegradable Packaging Materials: Past, Present, and Future (Brief Review). Polymers.

[17] Griffin G.J.L. (1974). Biodegradable Fillers in Thermoplastics. Adv. Chem..

[18] Shelenkov P.G., Pantyukhov P.V., Popov A.A. (2018). Highly Filled Biocomposites Based on Ethylene-Vinyl Acetate Copolymer and Wood Flour. IOP Conf. Ser. Mater. Sci. Eng..

[19] Shelenkov P.G., Pantyukhov P.V., Popov A.A. (2020). Mechanical Properties of Superconcentrates Based on Ethylene-Vinyl Acetate Copolymer and Microcrystalline Cellulose. Mater. Sci. Forum.

[20] Nikitin N.I. (1962). Chemistry of Wood and Cellulose.

[21] Fengel D., Wegener G. (1989). Wood—Chemistry, Ultrastructure, Reactions.

[22] Kuleznev V.N., Shershnev V.A. (1988). The Chemistry and Physics of Polymers.

[23] Zykova A.K., Pantyukhov P.V., Kolesnikova N.N., Monakhova T.V., Popov A.A. (2018). Influence of Filler Particle Size on Physical Properties and Biodegradation of Biocomposites Based on Low-Density Polyethylene and Lignocellulosic Fillers. J. Polym. Environ..

[24] Zaini M.J., Fuad M.Y.A., Ismail Z., Mansor M.S., Mustafah J. (1996). The Effect of Filler Content and Size on the Mechanical Properties of Polypropylene/Oil Palm Wood Flour Composites. Polym. Int..

[25] Mahdi E., Dean A. (2020). The Effect of Filler Content on the Tensile Behavior of Polypropylene/Cotton Fiber and Poly(Vinyl Chloride)/Cotton Fiber Composites. Materials.

