# Supplementary material for: Influence of Vinyl Acetate Content and Melt Flow Index of Ethylene-Vinyl Acetate Copolymer on Physico-Mechanical and Physico-Chemical Properties of Highly Filled Biocomposites
Source: Polymers (Basel). 2023 Jun 10;15(12):2639. doi: 10.3390/polym15122639 (PMC10304730; doi:10.3390/polym15122639)

EVA 28150 +MCC 50 %

Speed: 100.000 mm/min

Extrusion Grinev (Thickness: 1.0 mm) ; +21.8°C, 7.7%HR Test Data: 15-03-2018 Metod

Test No.	Material	Width	Thick ness	Max. Load	Yeild point	Max. Load	Break	Elastic modulus	Input Grip	Grip traversa	Comentary
		mm	mm	MPa	MPa	%	MPa	MPa	%	%	o
1	28150 + Cell. 50%	4.1	0.16	2.3	2.3	11.9	0.5	5.8	43.3	41.3	OK
2	28150 + Cell. 50%	4.1	0.16	1.6	1.6	10.1	0.5	0.3	20.0	18.4	NO
3	28150 + Cell. 50%	4.1	0.16	1.8	1.8	10.2	0.5	22.5	18.3	17.5	NO
4	28150 + Cell. 50%	4.1	0.16	1.1	1.1	9.9	0.3	20.2	26.7	26.8	NO (speed 25mm)
5	28150 + Cell. 50%	4.1	0.15	2.4	2.4	13.4	0.7	16.6	36.7	34.7	OK
---	---	---	---	---	---	---	---	---	---	---	---
Avera	---	4.1	0.16	1.8	1.8	11.1	0.5	13.1	29.0	27.7	---
SD(N)	---	0.0	0.00	0.5	0.5	1.4	0.1	8.6	9.6	9.2	---
CV%	---	0.0	2.53	25.9	25.9	12.2	25.3	65.6	33.2	33.2	---

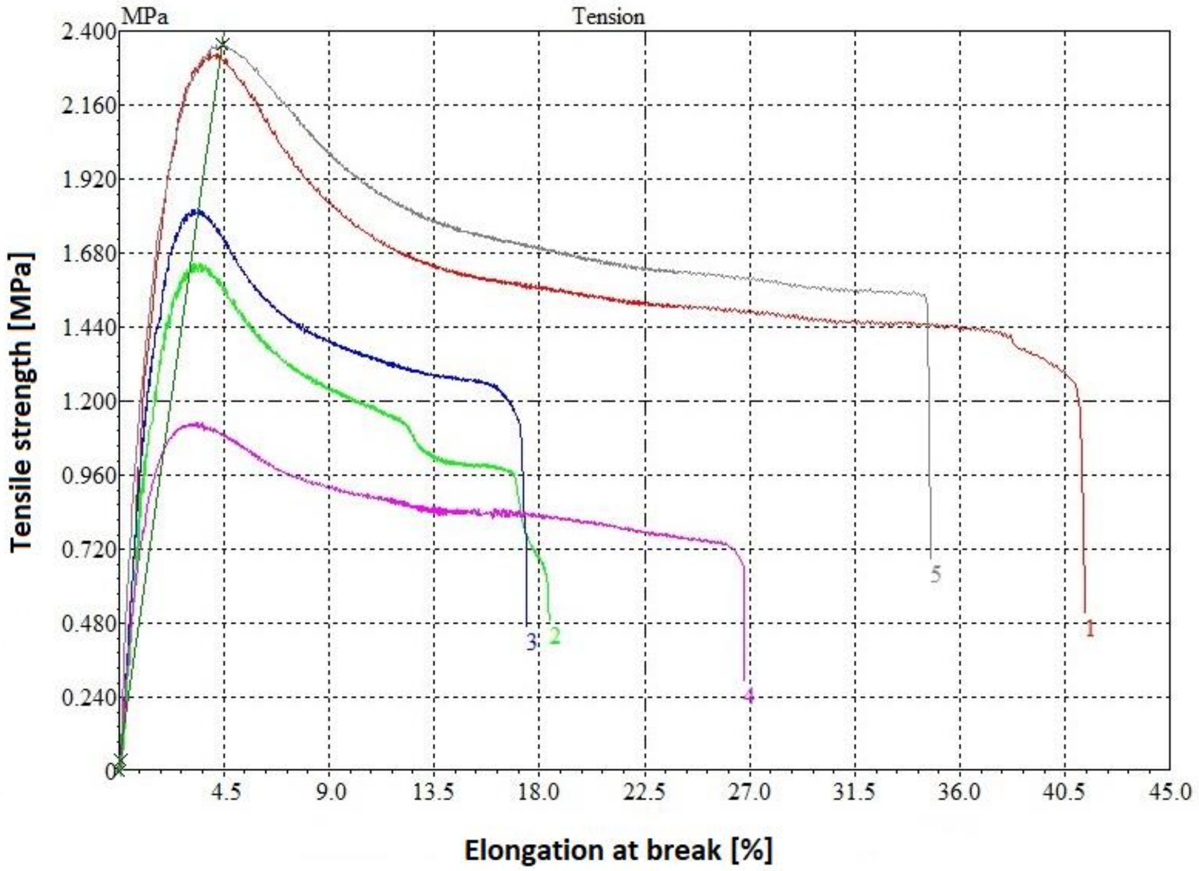

EVA 28025 + MCC 50%

Speed: 100.000 mm/min

Extrusion Grinev (Thickness: 1.0 mm) ; +21.8°C, 7.7%HR Test Data: 15-03-2018 Metod

Test No.	Material	Width mm	Thick ness mm	Max. Load MPa	Yeild point MPa	Max. Load %	Break MPa	Elastic modulus MPa	Input Grip %	Grip traversa %	Comentary º
1	28025 + Cell. 50%	4.1	0.20	3.0	3.0	4.3	0.5	115.5	186.7	186.2	OK
2	28025 + Cell. 50%	4.1	0.20	2.9	2.9	11.5	0.6	-	186.7	184.9	NO
3	28025 + Cell. 50%	4.1	0.21	3.1	3.1	12.7	0.4	15.2	170.0	167.7	No (d)
4	28025 + Cell. 50%	4.1	0.21	3.3	3.3	12.0	0.6	22.9	180.0	177.9	OK
5	28025 + Cell. 50%	4.1	0.19	3.3	3.3	14.0	0.5	22.5	185.0	183.7	OK
---	---	---	---	---	---	---	---	---	---	---	---
Avera	---	4.1	0.20	3.1	3.1	10.9	0.5	44.0	181.7	180.1	---
SD(N)	---	0.0	0.01	0.2	0.2	3.4	0.1	41.4	6.3	6.8	---
CV%	---	0.0	3.70	5.1	5.1	31.2	14.4	94.0	3.5	3.8	---

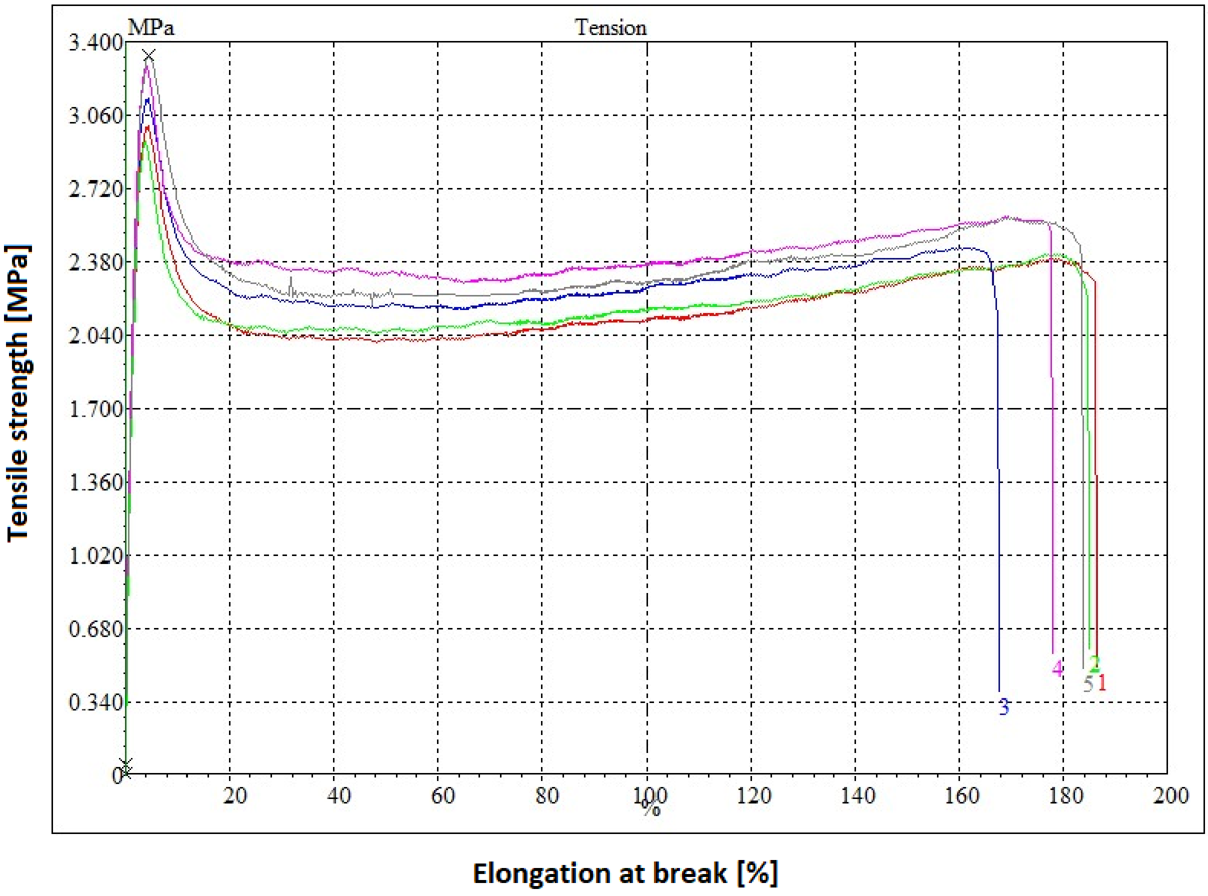

EVA 28005 + MCC 50%

Speed: 100.000 mm/min

Extrusion Grinev (Thickness: 1.0 mm) ; +21.8°C, 7.7%HR Test Data: 16-03-2018

Metod

Test No.	Material	Width mm	Thickness mm	Max. Load MPa	Yeild point MPa	Max. Load %	Break MPa	Elastic modulus MPa	Input Grip %	Grip traversa %	Comentary °
1	28005 + Cell. 50%	4.1	0.24	4.4	3.7	4.9	0.8	55.4	258.3	255.8	OK
2	28005 + Cell. 50%	4.1	0.25	3.8	3.4	4.7	0.7	64.6	233.3	230.3	OK
3	28005 + Cell. 50%	4.1	0.22	4.2	3.6	4.6	0.8	79.5	248.3	245.4	NO
4	28005 + Cell. 50%	4.1	0.23	4.2	3.9	4.6	0.7	131.8	225.0	222.2	NO
5	28005 + Cell. 50%	4.1	0.22	4.1	3.6	4.6	0.7	81.3	233.3	231.6	NO
---	---	---	---	---	---	---	---	---	---	---	---
Avera	---	4.1	0.23	4.1	3.6	4.7	0.7	82.5	239.6	237.1	---
SD(N)	---	0.0	0.01	0.2	0.2	0.1	0.0	26.4	12.0	12.0	---
CV%	---	0.0	5.03	4.7	4.5	2.5	6.6	32.0	5.0	5.1	---

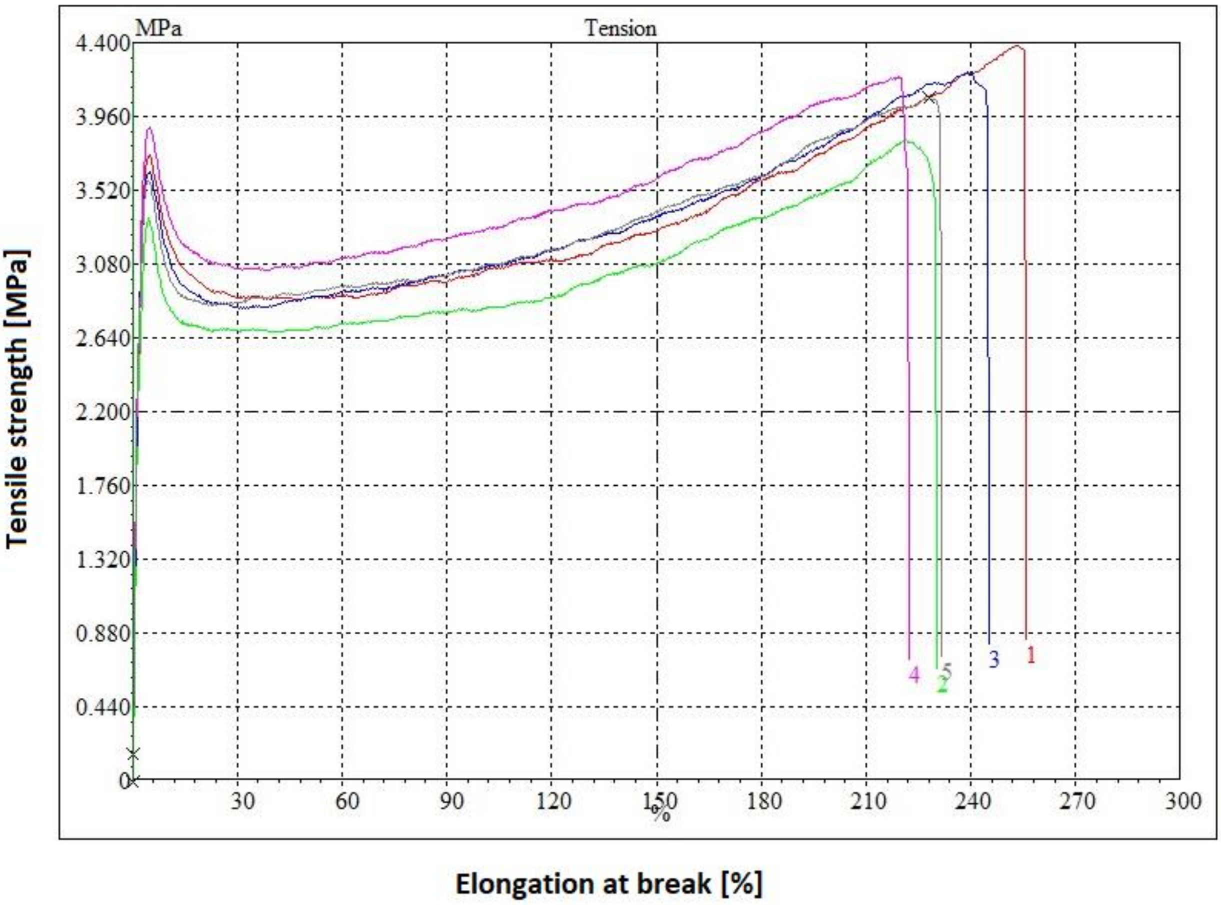

EVA 19150 + MCC 50%

Speed: 100.000 mm/min

Extrusion Grinev (Thickness: 1.0 mm) ; +21.8°C, 7.7%HR Test Data: 16-03-2018

Metod

Test No.	Material	Width	Thick ness	Max. Load	Yeild point	Max. Load	Break	Elastic modulus	Input Grip	Grip traversa	Comentary
		mm	mm	MPa	MPa	%	MPa	MPa	%	%	°
1	19150 + Cell. 50%	4.1	0.15	1.8	1.8	1.6	0.3	142.8	3.3	1.8	NO
2	19150 + Cell. 50%	4.1	0.14	3.0	3.0	2.1	0.6	212.2	5.0	3.3	NO
3	19150 + Cell. 50%	4.1	0.16	2.2	2.2	1.7	0.4	178.7	3.3	1.8	OK
4	19150 + Cell. 50%	4.1	0.15	2.1	2.1	1.7	0.4	149.9	3.3	2.2	NO
5	19150 + Cell. 50%	4.1	0.16	3.1	3.1	2.1	0.5	208.7	5.0	2.9	NO
---	---	---	---	---	---	---	---	---	---	---	---
Avera	---	4.1	0.15	2.4	2.4	1.8	0.4	178.5	4.0	2.4	---
SD(N)	---	0.0	0.01	0.5	0.5	0.2	0.1	28.8	0.8	0.6	---
CV%	---	0.0	4.92	21.2	21.2	11.7	23.2	16.1	20.9	25.1	---

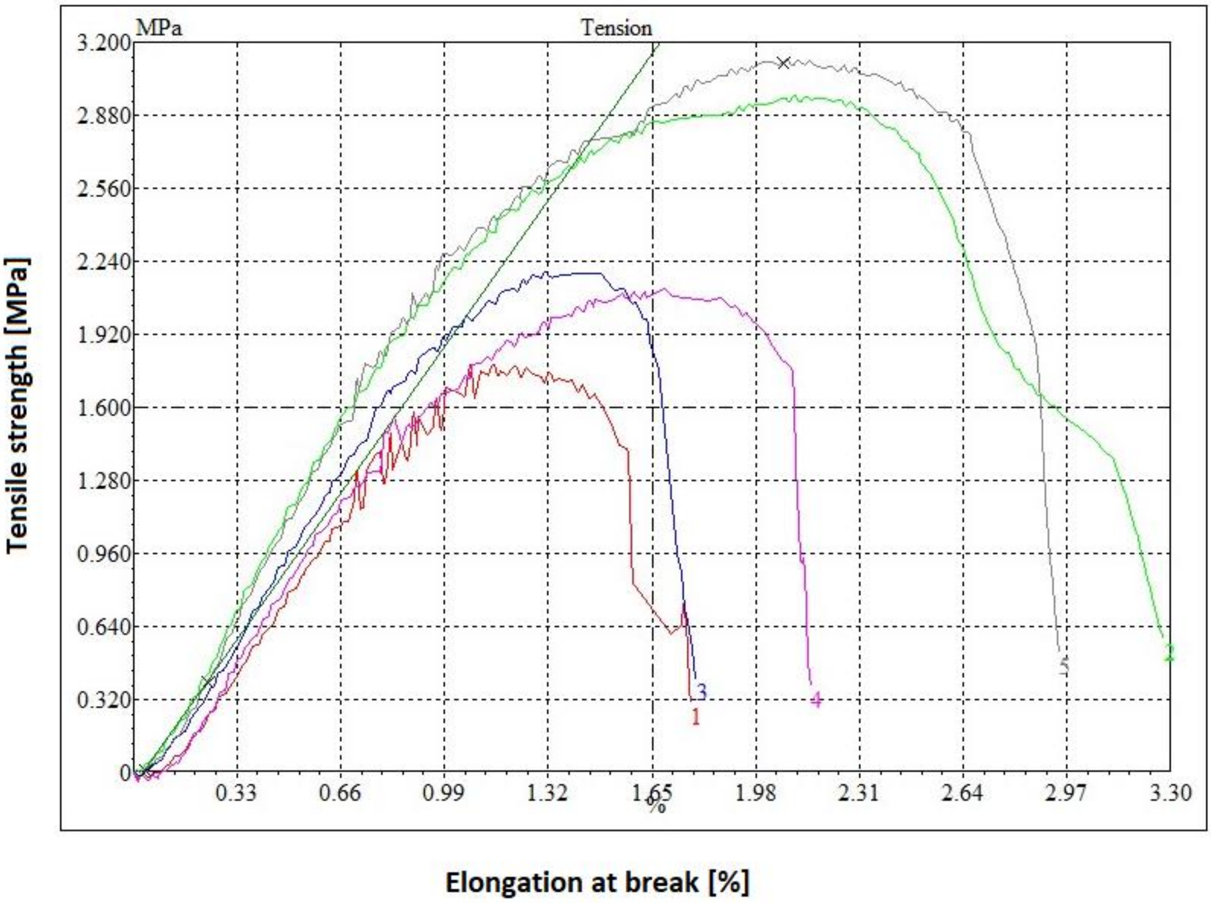

EVA 15006 + MCC 50%

Speed: 100.000 mm/min

Extrusion Grinev (Thickness: 1.0 mm) ; +21.8°C, 7.7%HR Test Data: 16-03-2018

Metod

Test No.	Material	Width mm	Thick ness mm	Max. Load MPa	Yeild point MPa	Max. Load %	Break MPa	Elastic modulus MPa	Input Grip %	Grip traversa %	Comentary o
1	15006 + Cell. 50%	4.1	0.28	4.7	4.7	2.3	0.9	269.9	10.0	8.3	OK
2	15006 + Cell. 50%	4.1	0.27	4.7	4.7	2.4	0.9	474.2	10.0	7.6	OK
3	15006 + Cell. 50%	4.1	0.27	4.9	4.9	2.3	1.0	240.6	8.3	6.4	NO
4	15006 + Cell. 50%	4.1	0.29	5.3	5.3	2.7	1.0	229.3	10.0	8.1	NO
~~5~~	~~15006 + Cell. 50%~~	~~4.1~~	~~0.25~~	~~0.1~~	~~0.1~~	-	~~0.1~~	-	~~10.0~~	~~0.6~~	~~NO~~
6	15006 + Cell. 50%	4.1	0.25	5.3	5.3	2.5	0.7	284.8	8.3	6.9	NO
Avera	---	4.1	0.27	5.0	5.0	2.4	0.9	299.8	9.3	7.5	---
SD(N)	---	0.0	0.01	0.3	0.3	0.1	0.1	89.5	0.8	0.7	---
CV%	---	0.0	4.88	5.4	5.4	6.1	12.2	29.8	8.9	9.6	---

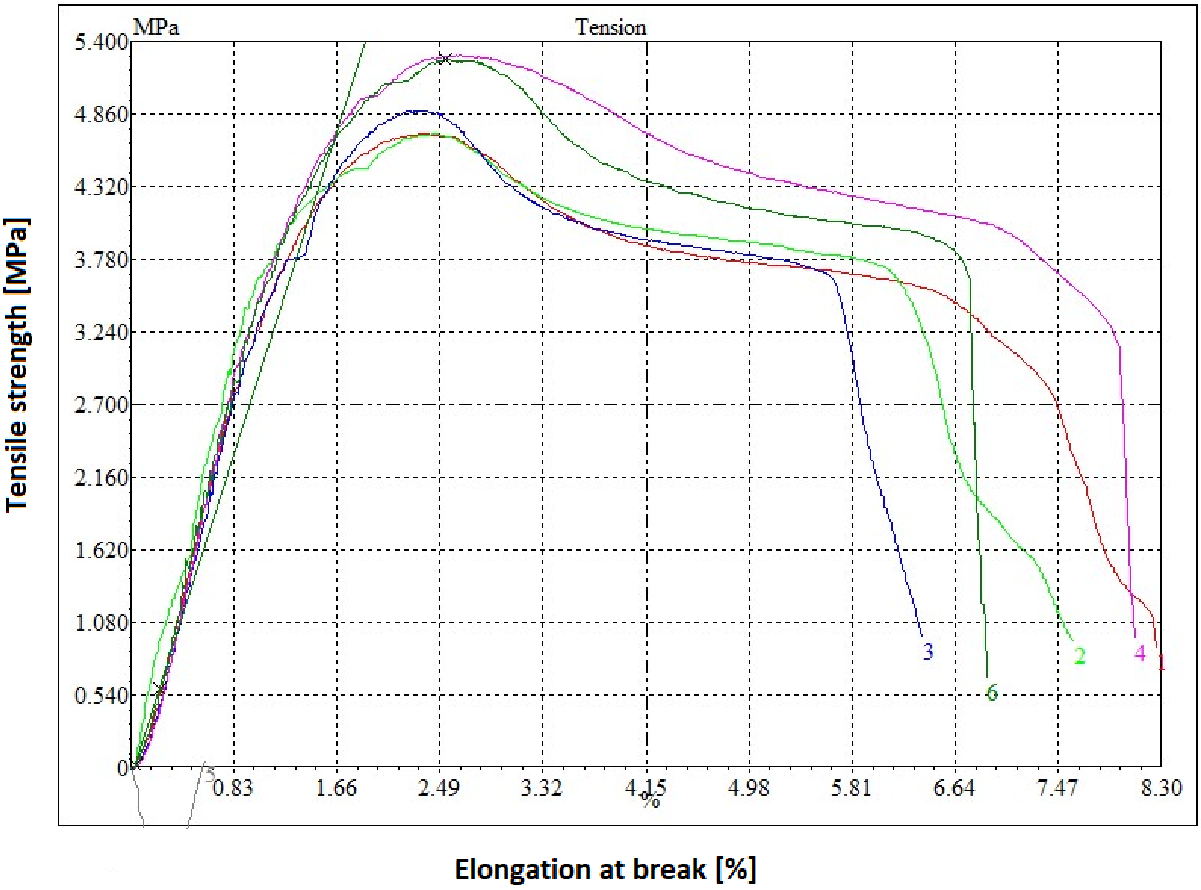

EVA 28150 + MCC 60%

Speed: 100.000 mm/min

Extrusion Grinev (Thickness: 1.0 mm) ; +21.8°C, 7.7%HR Test Data: 28-03-2018

Metod Extension #75

Test No.	Material	Width mm	Thick ness mm	Max. Load MPa	Yeild point MPa	Max. Load %	Break MPa	Elastic modulus MPa	Input Grip %	Grip traversa %	Comentary º
1	EVA 28150+Cell 60%	4.1	0.30	3.4	3.4	2.9	0.7	139.8	5.0	5.0	NO
2	EVA 28150+Cell 60%	4.1	0.31	3.3	3.3	2.6	0.6	281.0	3.3	4.2	NO
3	EVA 28150+Cell 60%	4.1	0.32	2.9	2.9	2.6	0.6	215.9	5.0	5.9	OK
4	EVA 28150+Cell 60%	4.1	0.32	3.3	3.3	2.4	0.6	267.1	3.3	4.0	NO
---	---	---	---	---	---	---	---	---	---	---	---
---	---	---	---	---	---	---	---	---	---	---	---
Avera	---	4.1	0.31	3.2	3.2	2.6	0.6	226.0	4.2	4.8	---
SD(N)	---	0.0	0.01	0.2	0.2	0.2	0.0	55.3	0.8	0.7	---
CV%	---	0.0	2.65	6.0	6.0	6.8	6.9	24.5	20.5	15.7	---

Tensile strength [MPa]

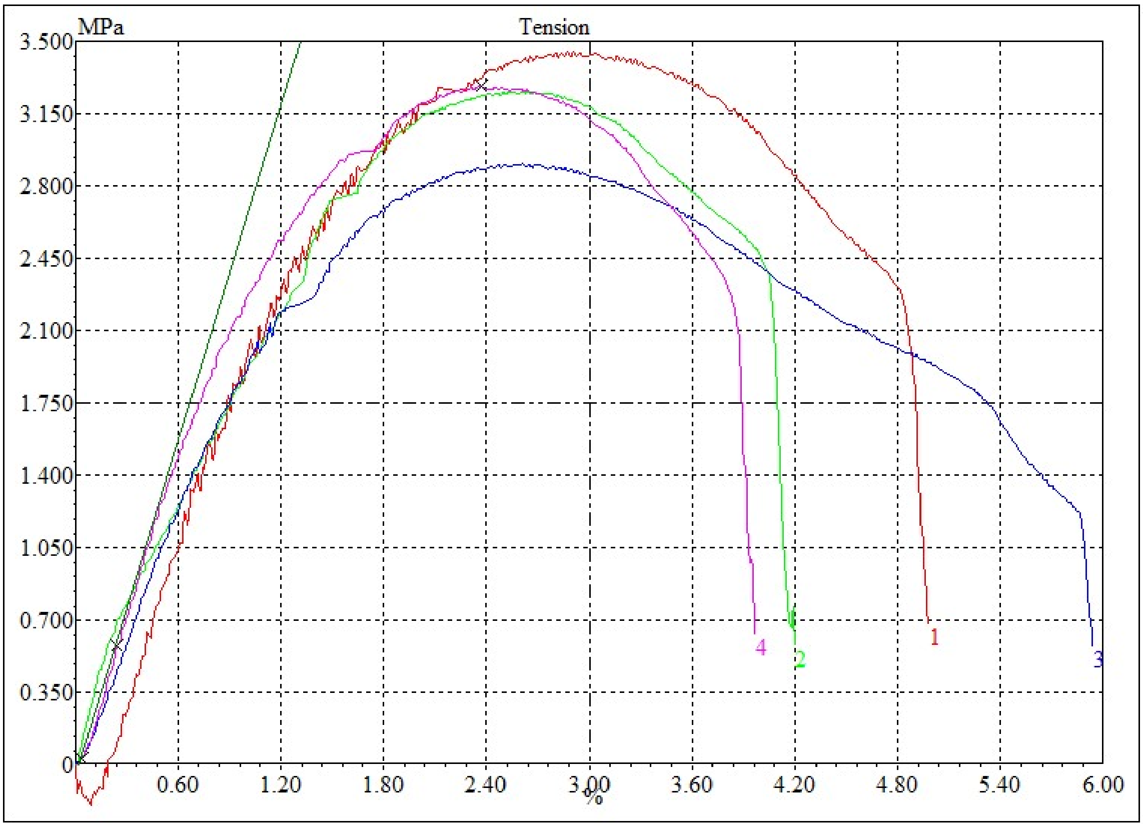

Elongation at break [%]

EVA 28025 + MCC 60%

Speed: 100.000 mm/min

Extrusion Grinev (Thickness: 1.0 mm) ; +21.8°C, 7.7%HR Test Data: 28-03-2018

Metod Extension #75

Test No.	Material	Width mm	Thick ness mm	Max. Load MPa	Yeild point MPa	Max. Load %	Break MPa	Elastic modulus MPa	Input Grip %	Grip traversa %	Comentary °
1	EVA 28025+Cell 60%	4.1	0.28	4.6	4.6	3.1	0.9	271.7	6.7	6.4	NO
2	EVA 28025+Cell 60%	4.1	0.29	4.4	4.4	3.1	0.8	270.1	8.3	9.4	OK
3	EVA 28025+Cell 60%	4.1	0.30	4.5	4.5	3.1	0.9	260.8	6.7	7.0	OK
---	---	---	---	---	---	---	---	---	---	---	---
---	---	---	---	---	---	---	---	---	---	---	---
---	---	---	---	---	---	---	---	---	---	---	---
Avera	---	4.1	0.29	4.5	4.5	3.1	0.9	267.5	7.2	7.6	---
SD(N)	---	0.0	0.01	0.1	0.1	0.0	0.0	4.8	0.8	1.3	---
CV%	---	0.0	2.82	1.8	1.8	0.0	5.4	1.8	10.4	17.1	---

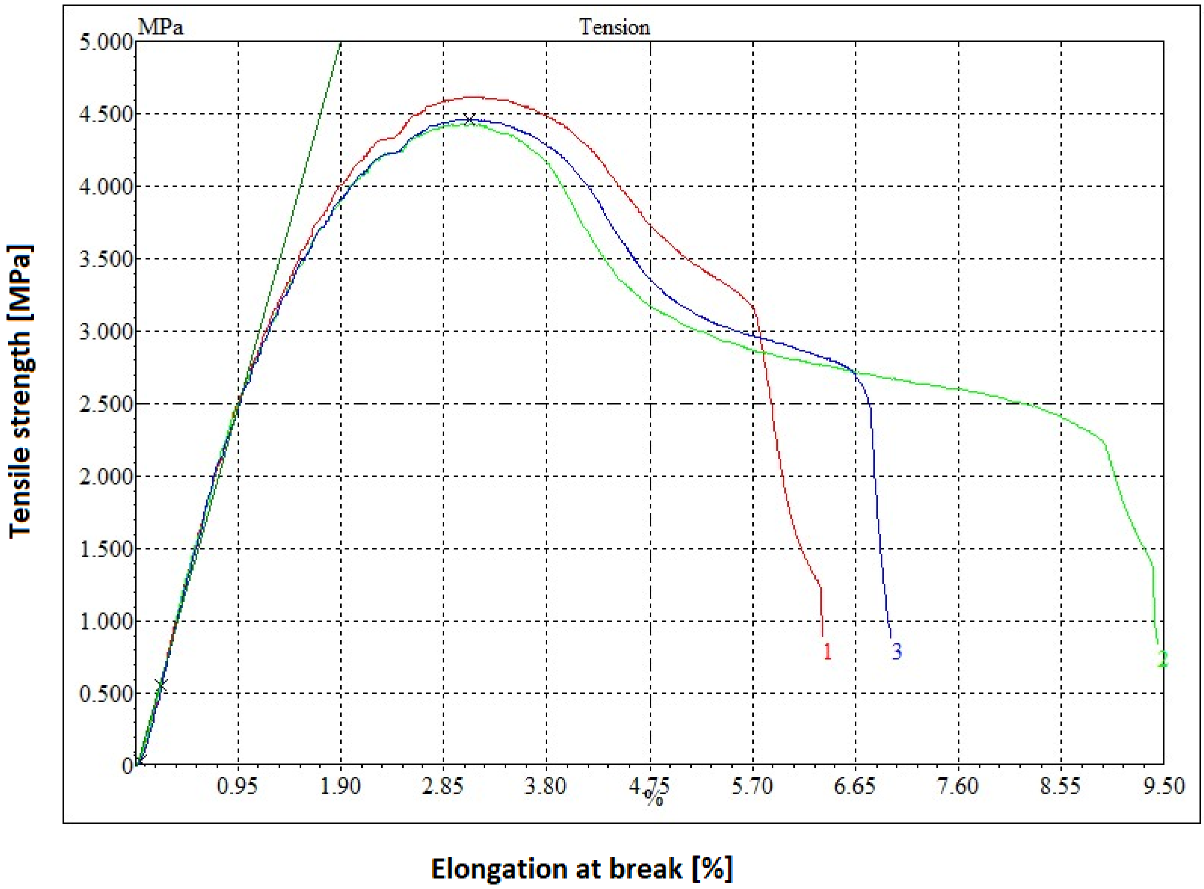

EVA 28005 + MCC 60%

Speed: 100.000 mm/min

Extrusion Grinev (Thickness: 1.0 mm) ; +21.8°C, 7.7%HR Test Data: 28-03-2018

Metod Extension #75

Test No.	Material	Width mm	Thick ness mm	Max. Load MPa	Yeild point MPa	Max. Load %	Break MPa	Elastic modulus MPa	Input Grip %	Grip traversa %	Comentary o
1	EVA 28005+Cell 60%	4.1	0.39	4.6	4.6	2.8	0.7	221.7	60.0	61.4	NO
2	EVA 28005+Cell 60%	4.1	0.34	4.7	4.7	2.7	0.9	395.8	23.3	24.1	NO
3	EVA 28005+Cell 60%	4.1	0.32	4.0	4.0	2.3	0.7	230.6	45.0	44.6	NO
---	---	---	---	---	---	---	---	---	---	---	---
---	---	---	---	---	---	---	---	---	---	---	---
---	---	---	---	---	---	---	---	---	---	---	---
Avera	---	4.1	0.35	4.4	4.4	2.6	0.8	282.7	42.8	43.4	---
SD(N)	---	0.0	0.03	0.3	0.3	0.2	0.1	80.1	15.1	15.3	---
CV%	---	0.0	8.41	7.0	7.0	8.3	12.3	28.3	35.2	35.2	---

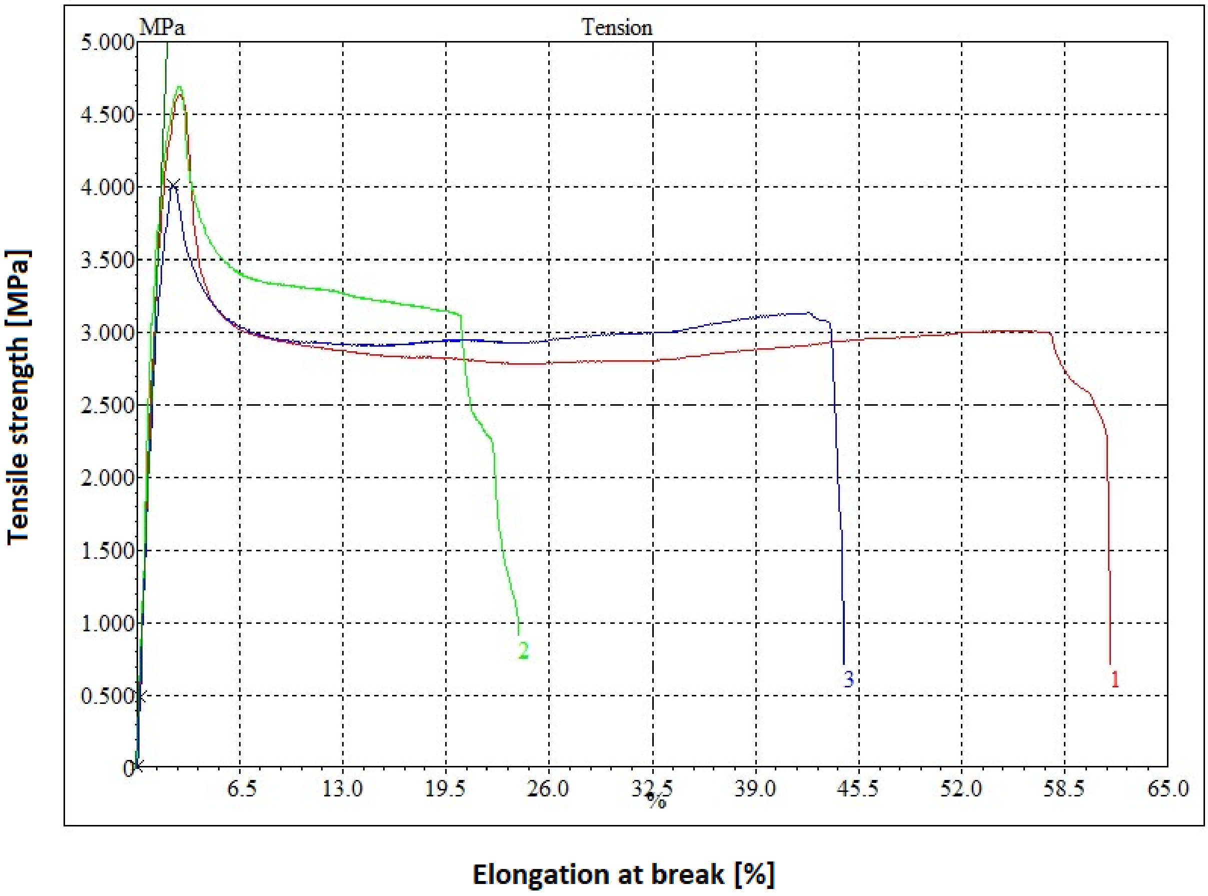

EVA 19150 + MCC 60%

Speed: 100.000 mm/min

Extrusion Grinev (Thickness: 1.0 mm) ; +21.8°C, 7.7%HR Test Data: 28-03-2018

Metod Extension #75

Test No.	Material	Width	Thick ness	Max. Load	Yeild point	Max. Load	Break	Elastic modulus	Input Grip	Grip traversa	Comentary
		mm	mm	MPa	MPa	%	MPa	MPa	%	%	o
1	EVA 19150+Cell 60%	4.1	0.23	5.6	5.6	1.7	1.0	599.5	2.5	2.3	NO
2	EVA 19150+Cell 60%	4.1	0.21	4.4	4.4	-	0.8	414.9	1.7	1.6	NO
3	EVA 19150+Cell 60%	4.1	0.23	5.4	5.4	1.7	0.8	589.3	1.7	1.8	NO
---	---	---	---	---	---	---	---	---	---	---	---
---	---	---	---	---	---	---	---	---	---	---	---
---	---	---	---	---	---	---	---	---	---	---	---
Avera	---	4.1	0.22	5.1	5.1	1.7	0.9	534.6	2.0	1.9	---
SD(N)	---	0.0	0.01	0.5	0.5	0.0	0.1	84.7	0.4	0.3	---
CV%	---	0.0	4.22	10.2	10.2	0.0	10.9	15.8	19.2	15.5	---

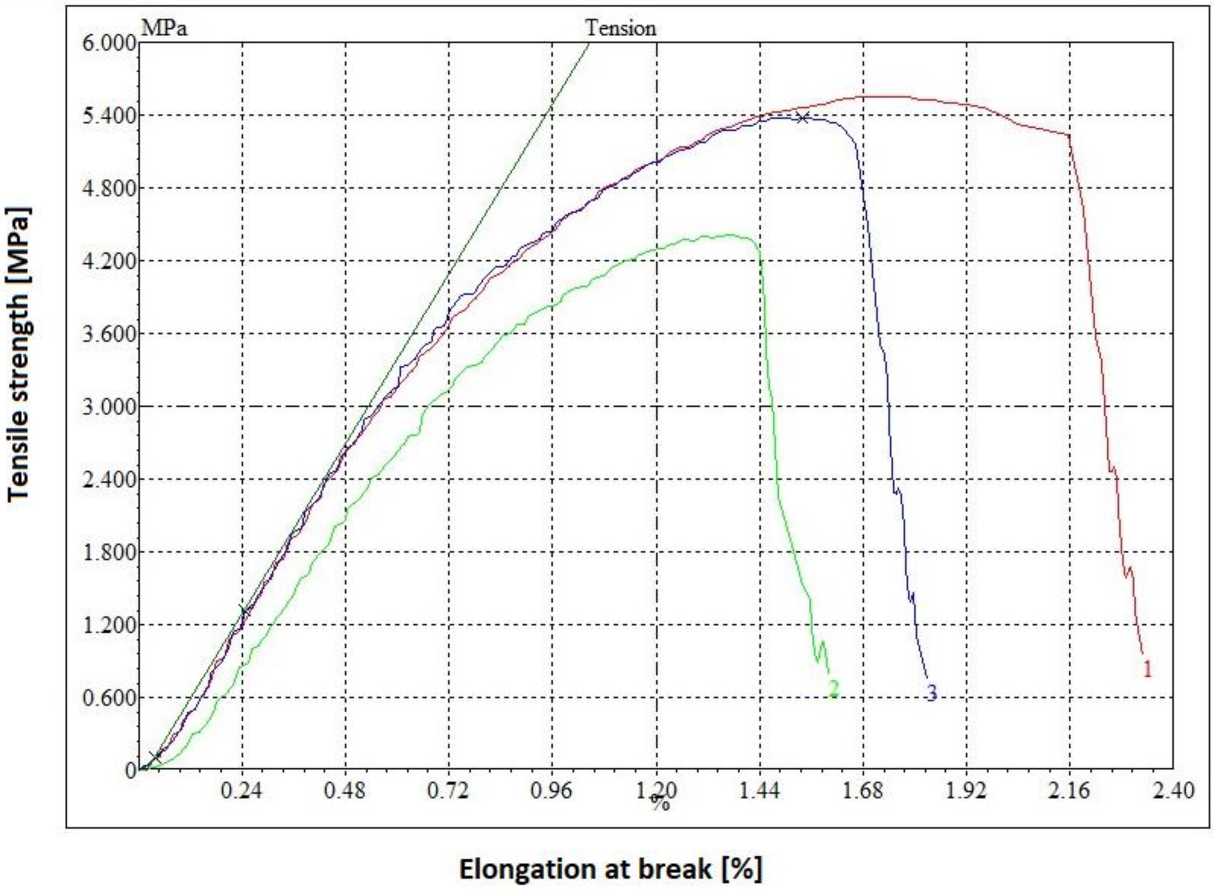

EVA 15006 + MCC 60%

Speed: 100.000 mm/min

Extrusion Grinev (Thickness: 1.0 mm) ; +21.8°C, 7.7%HR Test Data: 28-03-2018

Metod Extension #75

Test No.	Material	Width mm	Thick ness mm	Max. Load MPa	Yeild point MPa	Max. Load %	Break MPa	Elastic modulus MPa	Input Grip %	Grip traversa %	Comentary °
1	EVA 15006+Cell 60%	4.1	0.35	4.7	4.7	1.7	0.9	579.4	3.3	2.9	NO
2	EVA 15006+Cell 60%	4.1	0.32	5.4	5.4	1.7	0.8	460.7	1.7	2.8	NO
3	EVA 15006+Cell 60%	4.1	0.32	5.1	5.1	1.7	1.0	537.1	3.3	3.2	NO
---	---	---	---	---	---	---	---	---	---	---	---
---	---	---	---	---	---	---	---	---	---	---	---
---	---	---	---	---	---	---	---	---	---	---	---
Avera	---	4.1	0.33	5.1	5.1	1.7	0.9	525.7	2.8	3.0	---
SD(N)	---	0.0	0.01	0.3	0.3	0.0	0.1	49.1	0.8	0.2	---
CV%	---	0.0	4.29	5.7	5.7	0.0	9.1	9.3	27.3	5.7	---

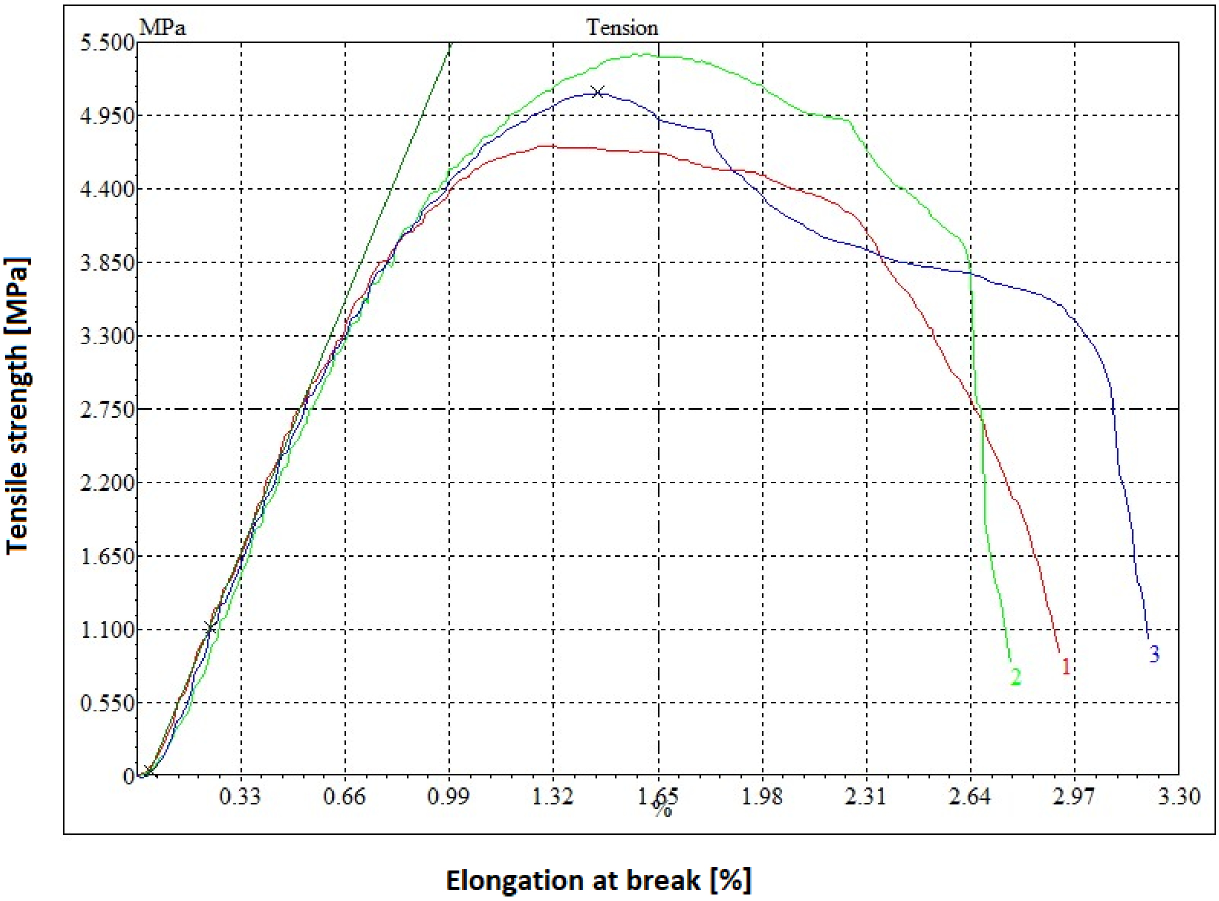

EVA 28150 + MCC 70%

Speed 100.000 mm/min

23°C, 35.5HR%, Test Data: 06-04-2018

Metod Extention Film

Test No.	Material			Width mm	Thick-ness mm	Max. Load N	Max. Load MPa	Yield point MPa	Elastic modulus MPa	Comments o
1	EVA	28150	Cell. (70%)	10.0	0.950	31.3	3.3	3.3	174.5	NO
2	EVA	28150	Cell. (70%)	10.0	0.940	29.8	3.2	3.2	91.6	NO
3	EVA	28150	Cell. (70%)	10.0	0.930	27.2	2.9	2.9	57.6	NO
4	EVA	28150	Cell. (70%)	10.0	0.850	19.6	2.3	2.3	176.0	Drugoi obrazec
---	---			---	---	---	---	---	---	---
Aver	---			10.0	0.917	27.0	2.9	2.9	124.9	---
SD(N)	---			0.0	0.040	4.5	0.4	0.4	51.7	---
CV%	---			0.0	4.317	16.7	13.3	13.3	41.4	---

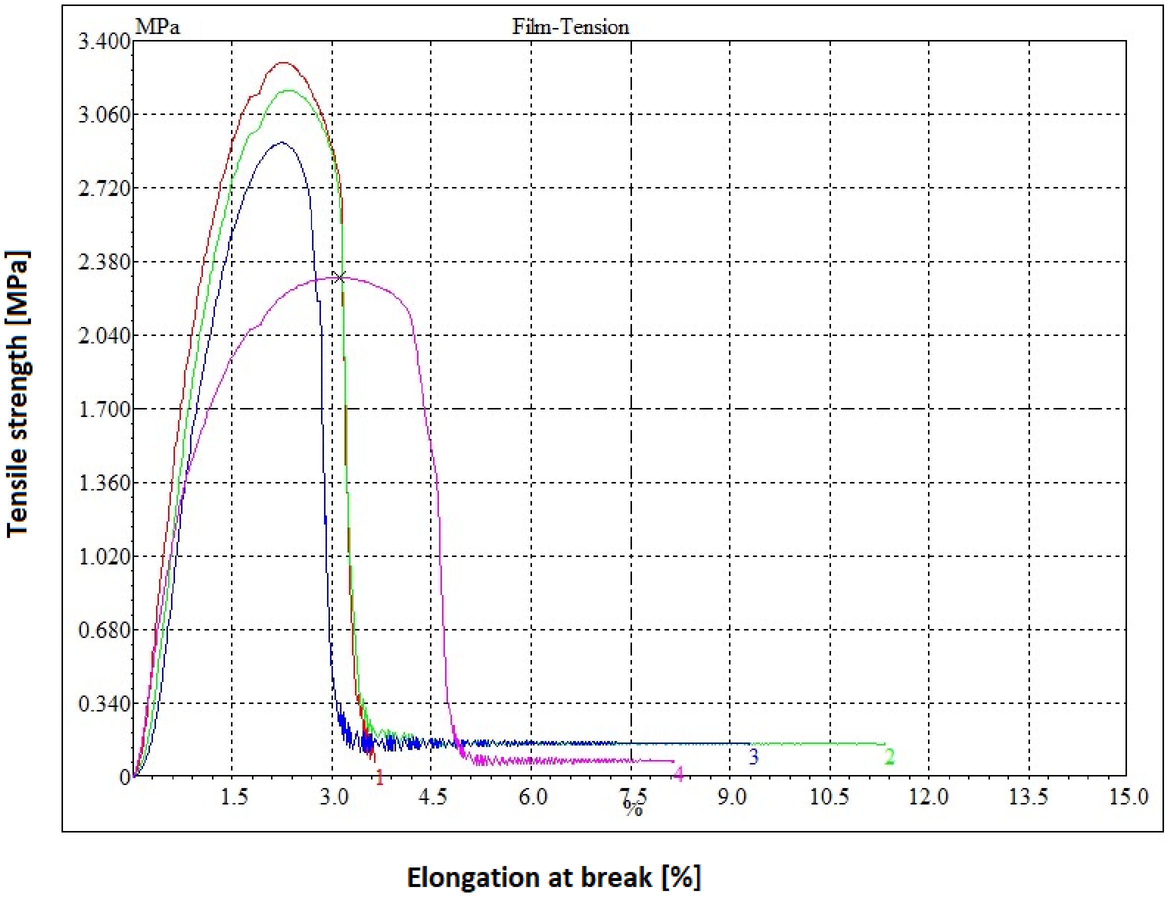

EVA 28025 + MCC 70%

Speed 100.000 mm/min

23°C, 26.5HR%, Test Data: 06-04-2018

Metod Extention Film

Test No.	Material	Width mm	Thick-ness mm	Max. Load N	Max. Load MPa	Yield point MPa	Elastic modulus MPa	Comments o
1	EVA 28025 + Cell. (70%)	10.0	0.800	29.8	3.7	3.7	91.1	63
2	EVA 28025 + Cell. (70%)	10.0	0.800	34.1	4.3	4.3	183.6	
3	EVA 28025 + Cell. (70%)	10.0	0.790	24.7	3.1	3.1	170.4	
4	EVA 28025 + Cell. (70%)	10.0	0.460	16.2	3.5	3.5	278.8	drugoi obrazec
Aver	---	10.0	0.713	26.2	3.6	3.6	181.0	---
SD(N)	---	0.0	0.146	6.7	0.4	0.4	66.6	---
CV%	---	0.0	20.469	25.4	11.9	11.9	36.8	---

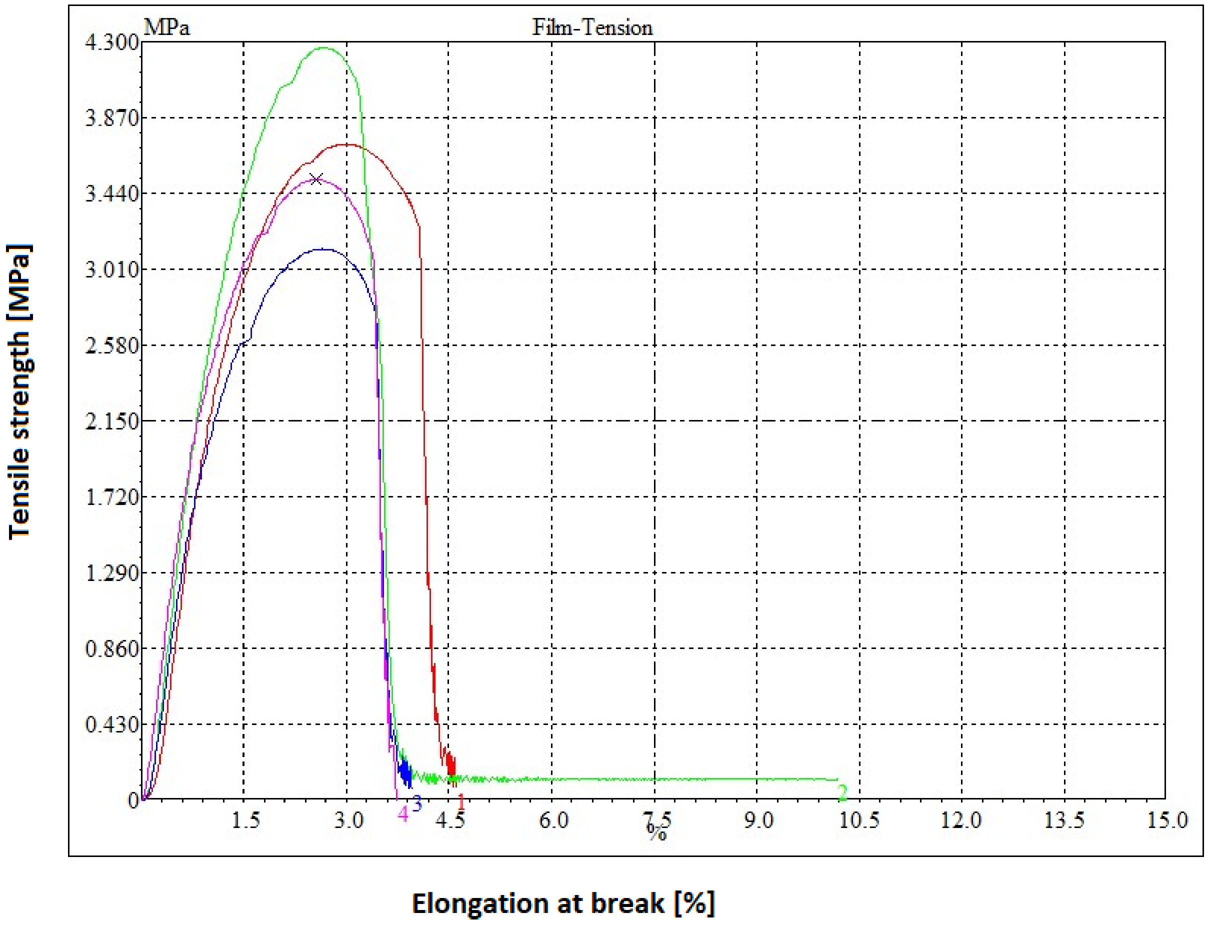

EVA 28005 + MCC 70%

Speed 100.000 mm/min

23°C, 26.5HR%, Test Data: 06-04-2018

Metod Extention Film

Test No.	Material	Width mm	Thick-ness mm	Max. Load N	Max. Load MPa	Yield point MPa	Elastic modulus MPa	Comments °
1	EVA 28005 + Cell. (70%)	10.0	0.440	18.6	4.2	4.2	70.2	ok
2	EVA 28005 + Cell. (70%)	10.0	0.560	25.9	4.6	4.6	330.1	ok
3	EVA 28005 + Cell. (70%)	10.0	0.450	19.9	4.4	4.4	183.1	ok
4	EVA 28005 + Cell. (70%)	10.0	0.400	17.3	4.3	4.3	255.5	drugoi
Aver	---	10.0	0.463	20.4	4.4	4.4	209.7	---
SD(N)	---	0.0	0.059	3.3	0.1	0.1	95.9	---
CV%	---	0.0	12.826	16.1	3.4	3.4	45.7	---

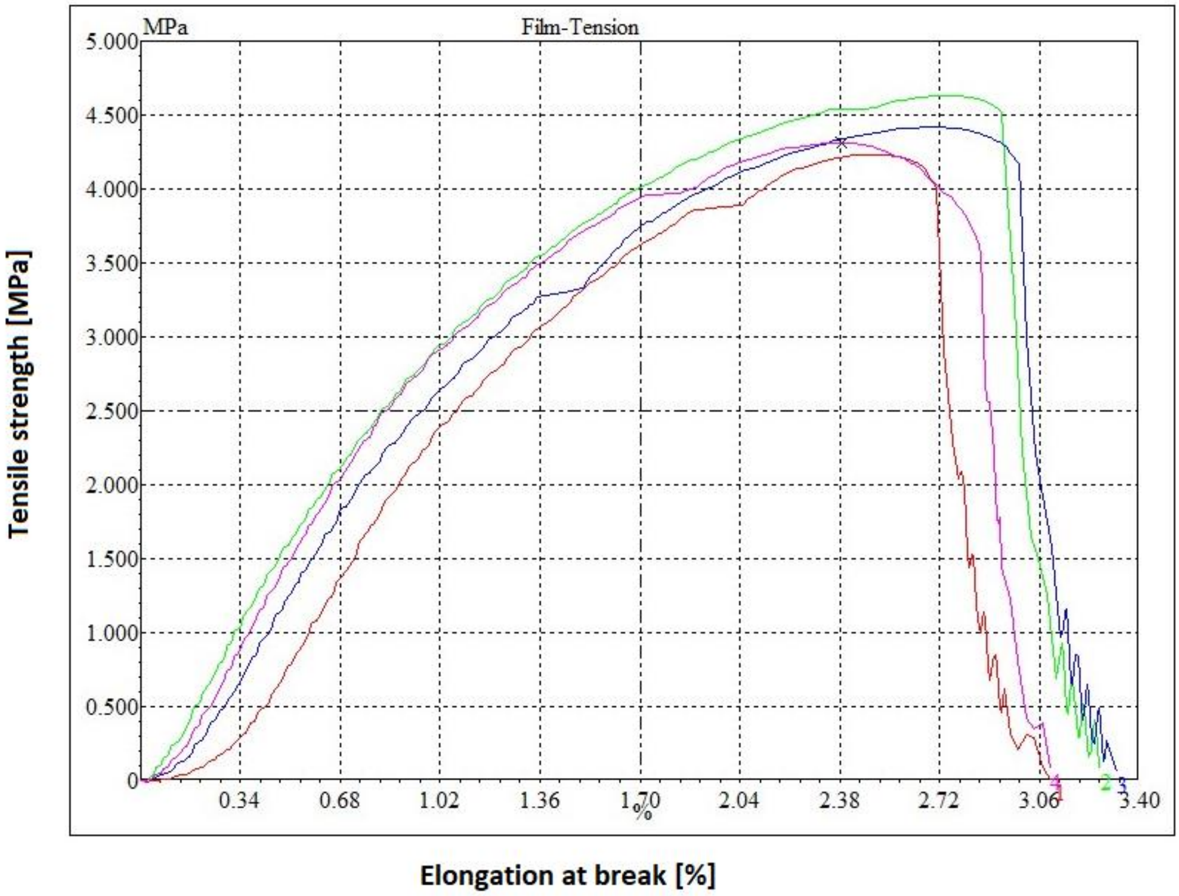

EVA 19150 + MCC 70%

23°C, 26.5HR%,   Test Data: 06-04-2018

Speed   100.000 mm/min

Metod   Extention Film

Test No.	Material	Width mm	Thick-ness mm	Max. Load N	Max. Load MPa	Yield point MPa	Elastic modulus MPa	Comments ο
1	EVA 19150 + Cell. (70%)	10.0	1.000	16.4	1.6	1.5	121.9	no
2	EVA 19150 + Cell. (70%)	10.0	1.000	31.0	3.1	3.1	151.9	
3	EVA 19150 + Cell. (70%)	10.0	0.860	12.1	1.4	1.4	233.8	Drugoi obrazec
4	EVA 19150 + Cell. (70%)	10.0	0.910	14.0	1.5	1.4	116.7	
Aver	---	10.0	0.943	18.4	1.9	1.9	156.1	---
SD(N)	---	0.0	0.060	7.4	0.7	0.7	46.8	---
CV%	---	0.0	6.383	40.5	36.7	39.1	30.0	---

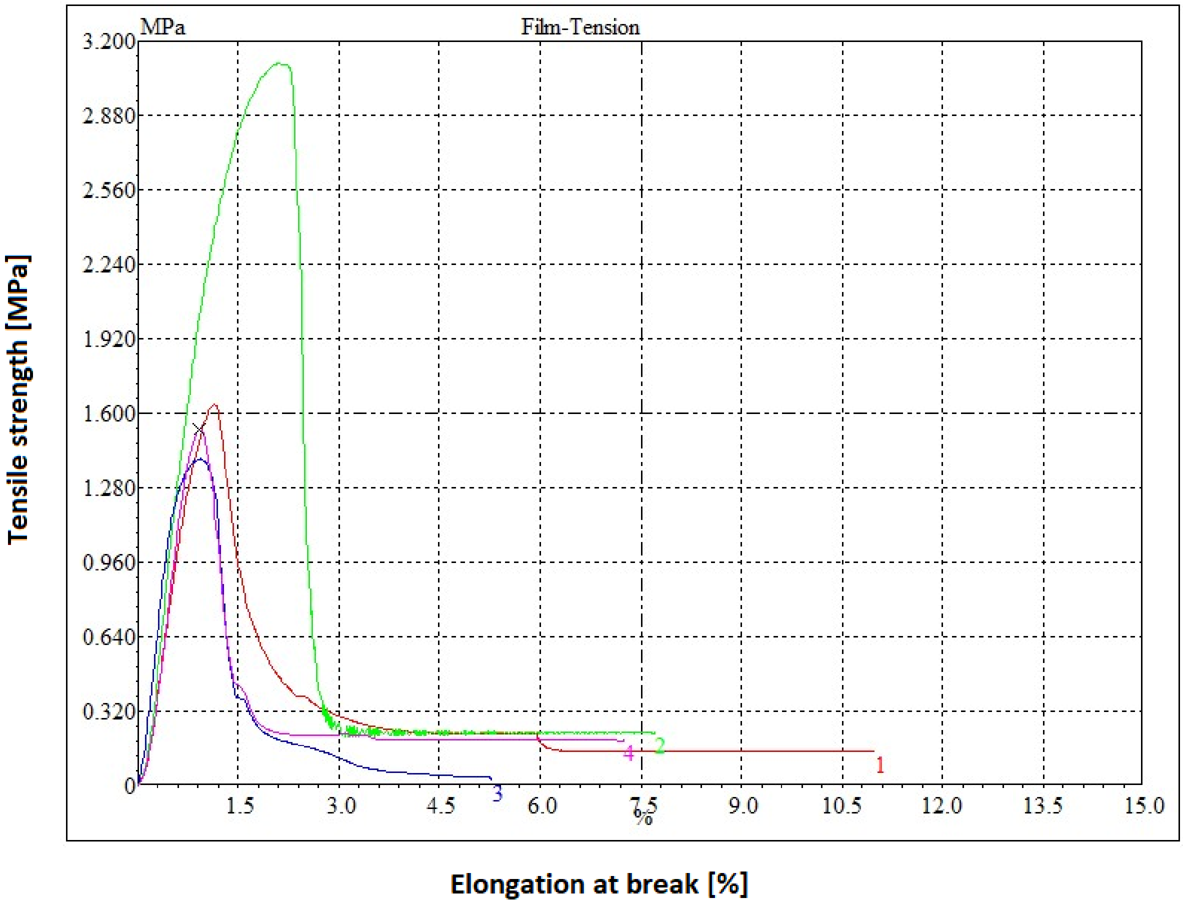

EVA 15006 + MCC 70%

Speed 100.000 mm/min

23°C, 26.5HR%, Test Data: 06-04-2018

Metod Extention Film

Test No.	Material	Width mm	Thick-ness mm	Max. Load N	Max. Load MPa	Yield point MPa	Elastic modulus MPa	Comments °
1	EVA 15006 + Cell. (70%)	10.0	0.540	32.9	6.1	5.7	70.3	
2	EVA 15006 + Cell. (70%)	10.0	0.570	25.7	4.5	4.3	85.3	
3	EVA 15006 + Cell. (70%)	10.0	0.530	29.3	5.5	5.1	205.6	
4	EVA 15006 + Cell. (70%)	10.0	0.440	16.4	3.7	3.6	428.6	Drugoi obrazec
Aver	---	10.0	0.520	26.1	5.0	4.7	197.4	---
SD(N)	---	0.0	0.048	6.1	0.9	0.8	143.4	---
CV%	---	0.0	9.322	23.5	18.6	17.0	72.6	---

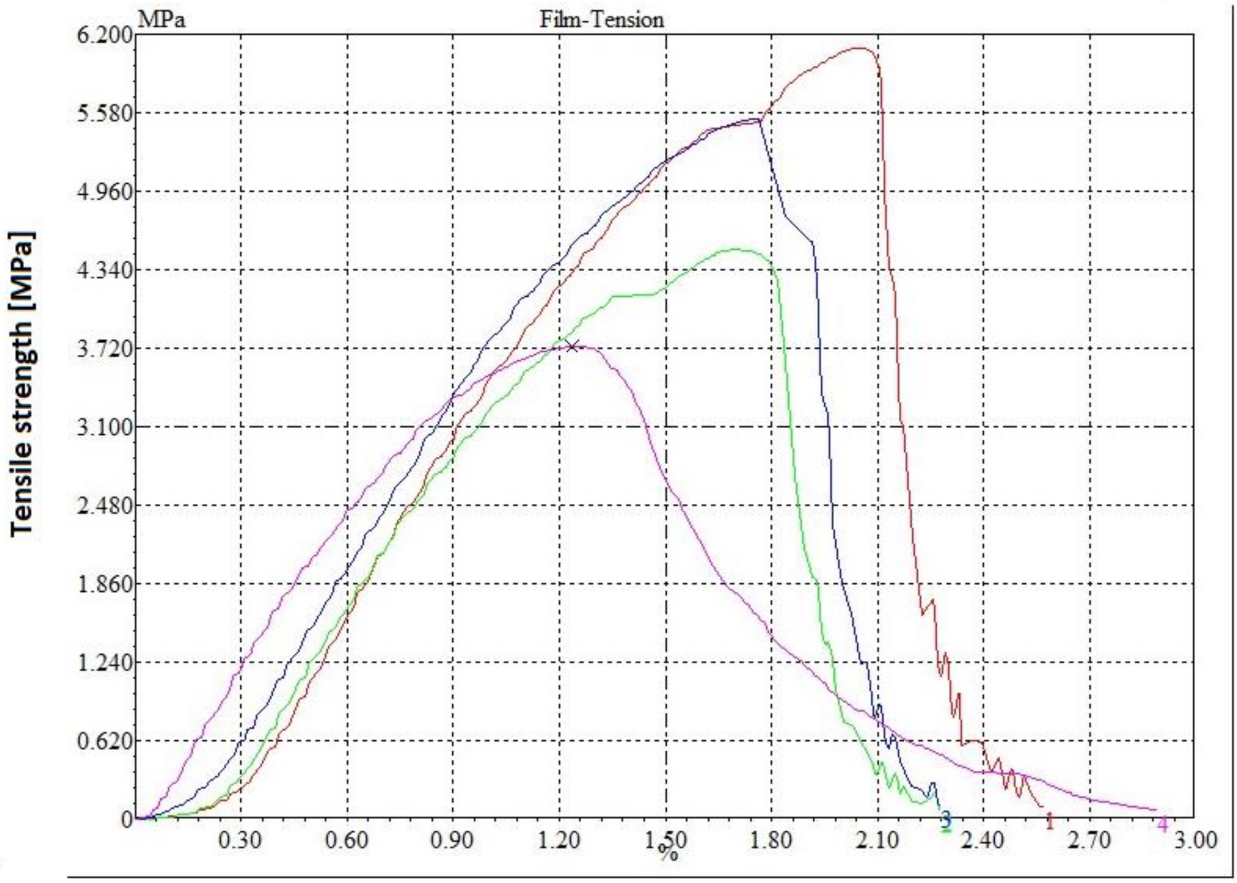

Elongation at break [%]

EVA 28150 + WF 50%

Speed: 100.000 mm/min

Extrusion Grinev (Thickness: 1.0 mm) ; +21.8°C, 7.7%HR Test Data: 05-12-2017

Metod Extension#75

Test No.	Material	Width mm	Thick ness mm	Max. Load MPa	Yeild point MPa	Max. Load %	Break MPa	Elastic modulus MPa	Input Grip %	Grip traversa %	Comentary °
1	EVA 28150 + W.F. (50%)	4.1	0.41	2.3	2.3	2.8	0.6	192.1	6.7	6.2	OK
2	EVA 28150 + W.F. (50%)	4.1	0.36	2.6	2.6	2.8	0.8	192.5	11.7	10.8	NO
3	EVA 28150 + W.F. (50%)	4.1	0.38	2.5	2.5	3.0	0.7	197.6	16.7	16.4	NO
4	EVA 28150 + W.F. (50%)	4.1	0.35	2.5	2.5	2.7	0.7	179.1	15.0	16.1	OK
---	---	---	---	---	---	---	---	---	---	---	---
---	---	---	---	---	---	---	---	---	---	---	---
Avera	---	4.1	0.38	2.5	2.5	2.8	0.7	190.3	12.5	12.4	---
SD(N)	---	0.0	0.02	0.1	0.1	0.1	0.1	6.8	3.8	4.2	---
CV%	---	0.0	6.11	4.4	4.4	3.9	10.1	3.6	30.4	34.0	---

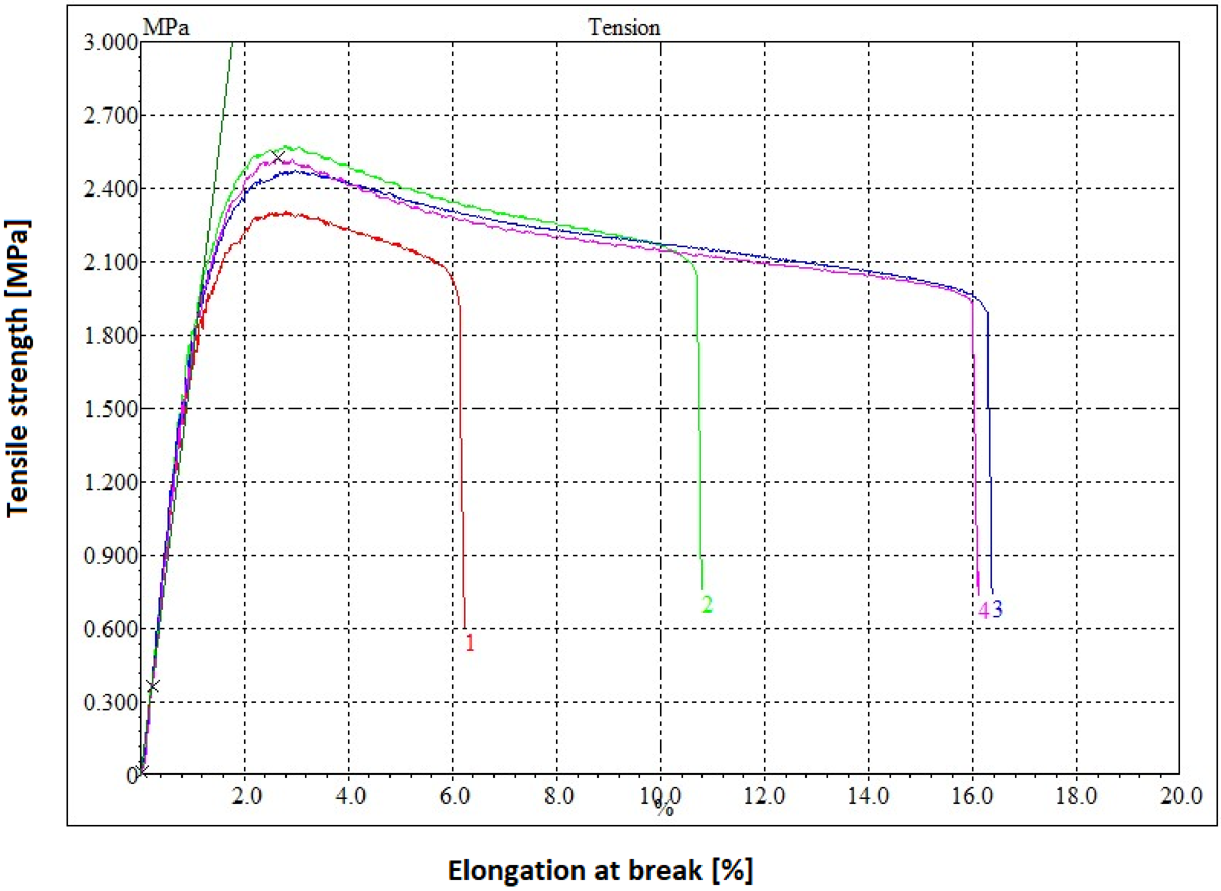

EVA 28025 + W.F. (50%)

Speed: 100.000 mm/min

Extrusion Grinev (Thickness: 1.0 mm) ; +21.8°C, 7.7%HR Test Data: 05-12-2017

Metod Extension#75

Test No.	Material	Width	Thick ness	Max. Load	Yeild point	Max. Load	Break	Elastic modulus	Input Grip	Grip traversa	Comentary
		mm	mm	MPa	MPa	%	MPa	MPa	%	%	°
1	EVA 28025 + W.F. (50%)	4.1	0.32	3.1	3.1	3.2	0.9	219.0	28.3	27.1	OK
2	EVA 28025 + W.F. (50%)	4.1	0.33	2.9	2.9	2.8	0.8	217.4	20.0	20.1	OK
3	EVA 28025 + W.F. (50%)	4.1	0.32	2.9	2.9	3.2	0.9	228.0	33.3	32.2	OK
4	EVA 28025 + W.F. (50%)	4.1	0.33	2.9	2.9	3.2	0.7	192.9	30.0	28.2	OK
---	---	---	---	---	---	---	---	---	---	---	---
---	---	---	---	---	---	---	---	---	---	---	---
Avera	---	4.1	0.33	3.0	3.0	3.1	0.8	214.3	27.9	26.9	---
SD(N)	---	0.0	0.01	0.1	0.1	0.2	0.1	13.0	4.9	4.4	---
CV%	---	0.0	1.54	2.9	2.9	5.6	10.1	6.1	17.6	16.2	---

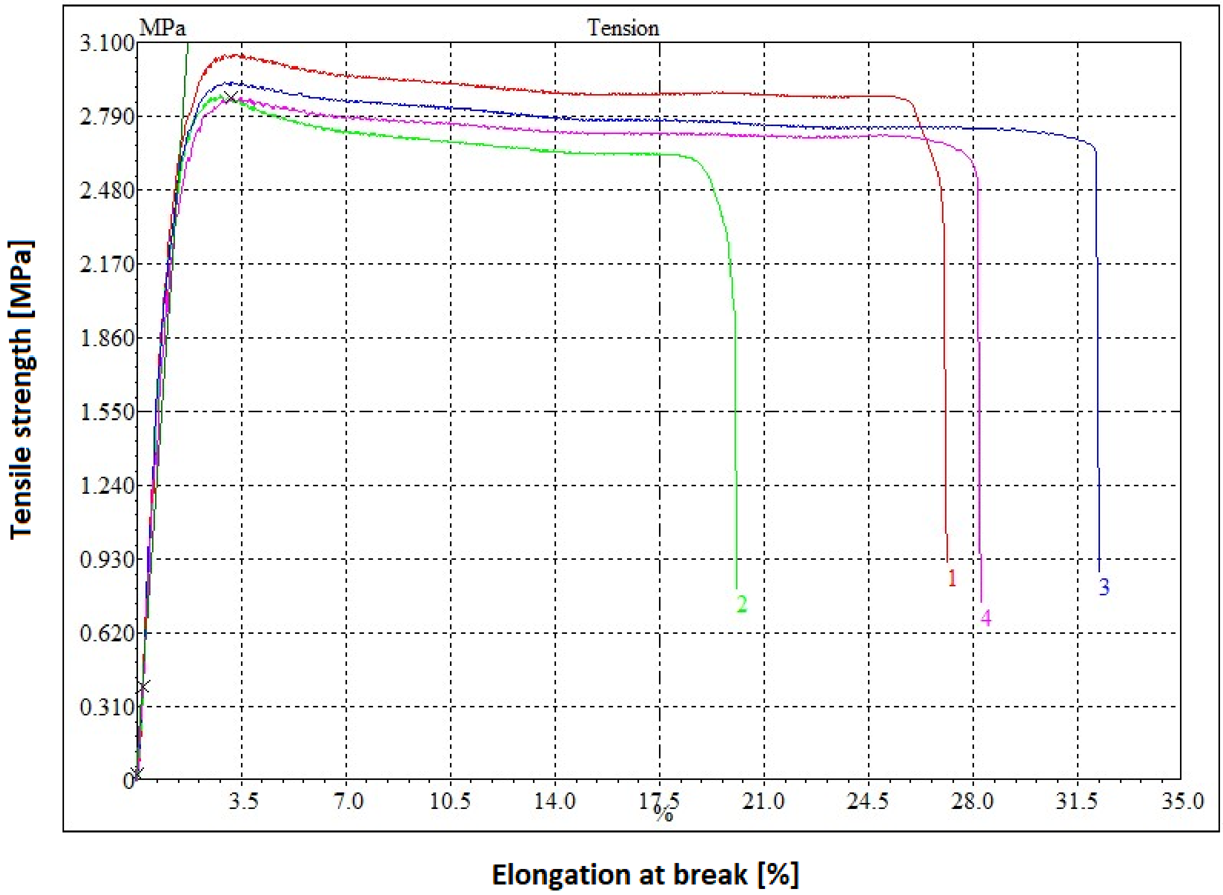

EVA 28005 + W.F. (50%)

Speed: 100.000 mm/min

Extrusion Grinev (Thickness: 1.0 mm) ; +21.8°C, 7.7%HR Test Data: 05-12-2017

Metod Extension#75

Test No.	Material	Width mm	Thick ness mm	Max. Load MPa	Yeild point MPa	Max. Load %	Break MPa	Elastic modulus MPa	Input Grip %	Grip traversa %	Comentary °
1	EVA 28005 + W.F. (50%)	4.1	0.31	3.2	3.1	33.2	0.8	198.5	60.0	57.5	OK
2	EVA 28005 + W.F. (50%)	4.1	0.31	3.4	3.4	4.1	0.9	247.6	55.0	54.8	OK
3	EVA 28005 + W.F. (50%)	4.1	0.27	3.4	3.4	3.6	1.0	260.5	50.0	50.1	NO
4	EVA 28005 + W.F. (50%)	4.1	0.33	3.6	3.6	4.3	1.0	246.0	53.3	52.4	OK
---	---	---	---	---	---	---	---	---	---	---	---
---	---	---	---	---	---	---	---	---	---	---	---
Avera	---	4.1	0.30	3.4	3.4	11.3	0.9	238.2	54.6	53.7	---
SD(N)	---	0.0	0.02	0.1	0.2	12.6	0.1	23.6	3.6	2.8	---
CV%	---	0.0	7.15	4.2	5.3	111.9	9.0	9.9	6.6	5.1	---

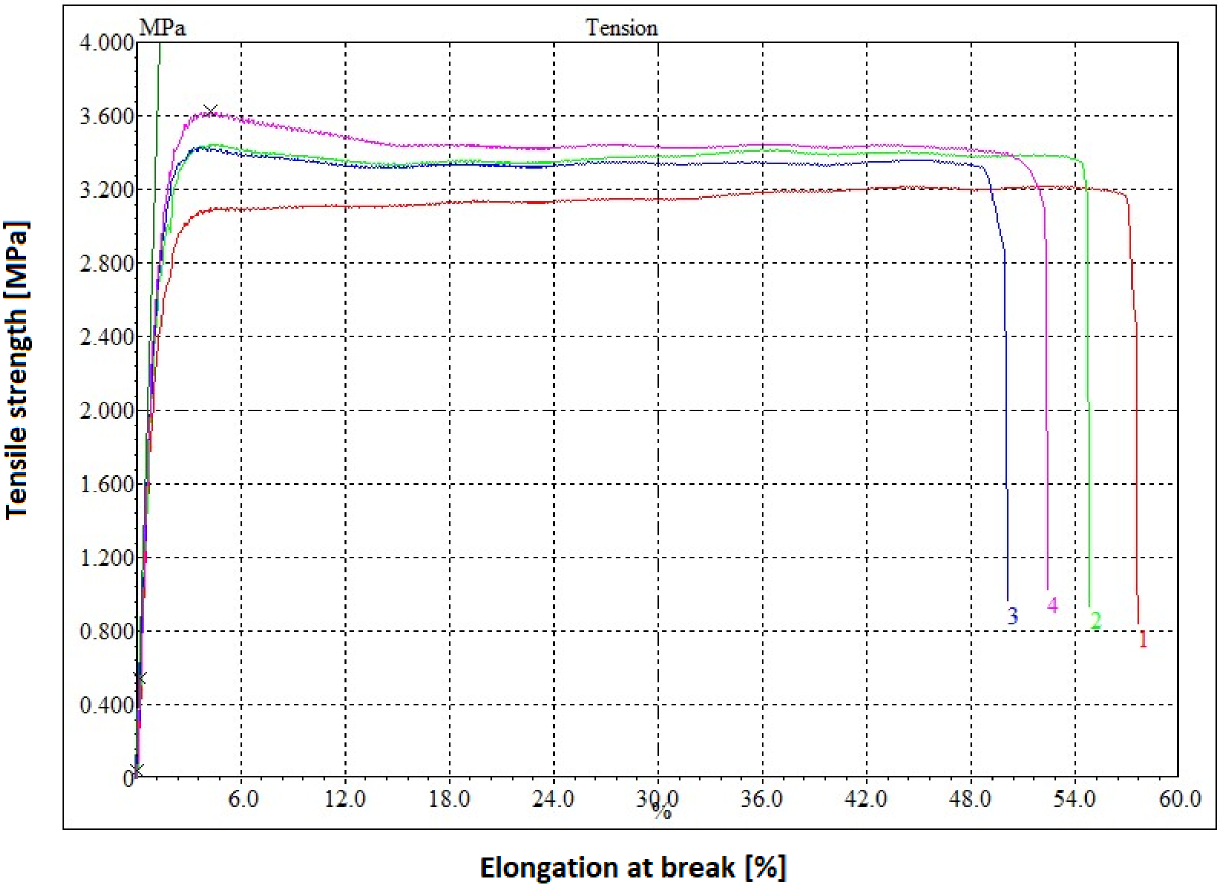

EVA 19150 + W.F. (50%)

Speed: 100.000 mm/min

Extrusion Grinev (Thickness: 1.0 mm) ; +21.8°C, 7.7%HR Test Data: 06-12-2017

Metod Extension#75

Test No.	Material	Width mm	Thick ness mm	Max. Load MPa	Yeild point MPa	Max. Load %	Break MPa	Elastic modulus MPa	Input Grip %	Grip traversa %	Comentary º
1	EVA 19150 + W.F. (50%)	4.1	0.29	3.0	3.0	2.2	0.8	292.9	5.0	4.1	OK
2	EVA 19150 + W.F. (50%)	4.1	0.29	3.1	3.1	2.3	0.8	336.6	3.3	3.7	NO
3	EVA 19150 + W.F. (50%)	4.1	0.30	3.3	3.3	2.4	0.9	275.2	5.0	3.9	OK
4	EVA 19150 + W.F. (50%)	4.1	0.30	3.0	3.0	2.3	0.7	316.8	5.0	4.1	NO
---	---	---	---	---	---	---	---	---	---	---	---
---	---	---	---	---	---	---	---	---	---	---	---
Avera	---	4.1	0.29	3.1	3.1	2.3	0.8	305.4	4.6	3.9	---
SD(N)	---	0.0	0.00	0.1	0.1	0.1	0.1	23.3	0.7	0.2	---
CV%	---	0.0	1.69	4.0	4.0	3.1	8.8	7.6	16.1	4.2	---

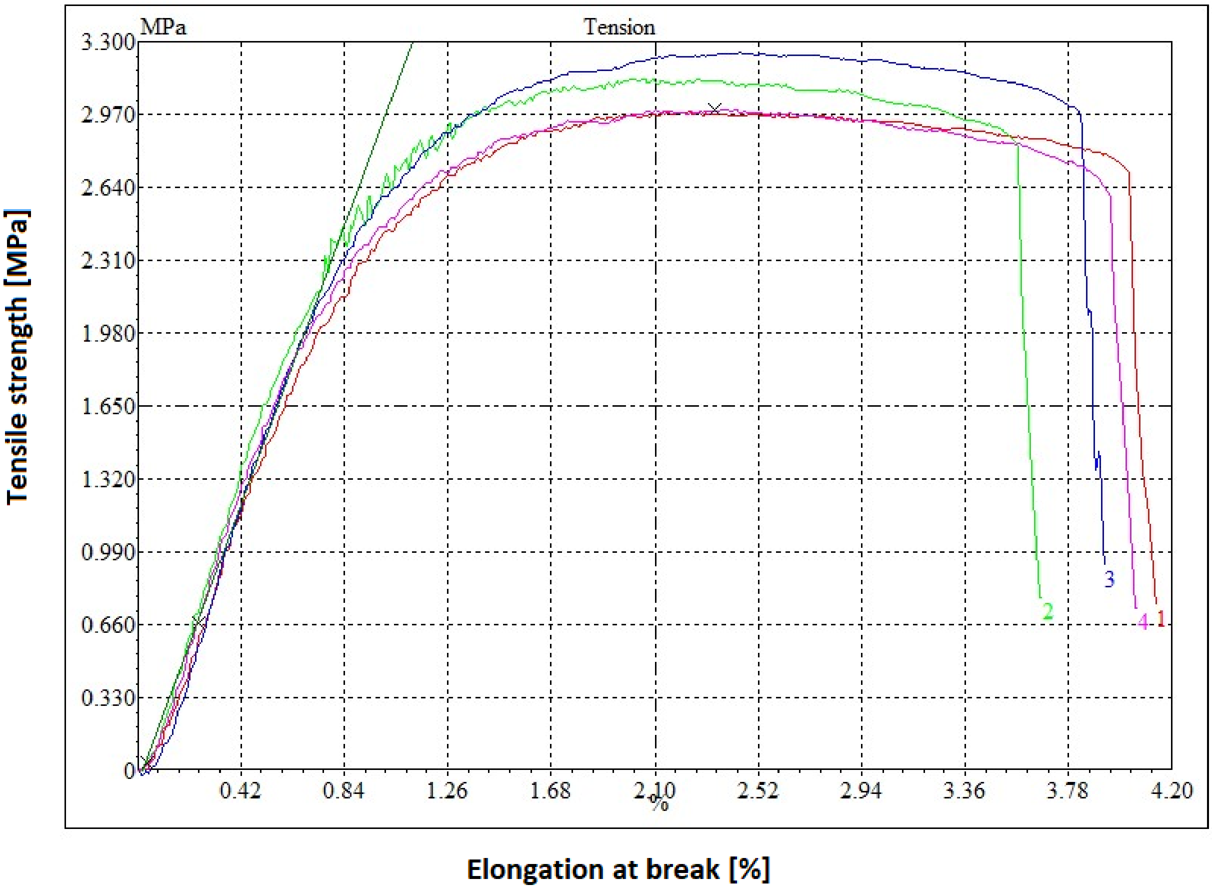

EVA 15006 + W.F. (50%) Speed: 100.000 mm/min

Extrusion Grinev (Thickness: 1.0 mm) ; +21.8°C, 7.7%HR Test Data: 06-12-2017 Metod Extension#75

Test No.	Material	Width mm	Thick ness mm	Max. Load MPa	Yeild point MPa	Max. Load %	Break MPa	Elastic modulus MPa	Input Grip %	Grip traversa %	Comentary °
1	EVA 15006 + W.F. (50%)	4.1	0.28	4.1	4.1	2.1	1.1	464.3	6.7	4.8	OK
2	EVA 15006 + W.F. (50%)	4.1	0.29	3.7	3.7	2.3	1.1	267.9	5.0	4.0	NO
3	EVA 15006 + W.F. (50%)	4.1	0.28	4.2	4.2	2.1	0.9	437.4	5.0	5.6	NO
4	EVA 15006 + W.F. (50%)	4.1	0.28	4.5	4.5	2.2	1.3	434.2	3.3	4.5	NO
---	---	---	---	---	---	---	---	---	---	---	---
---	---	---	---	---	---	---	---	---	---	---	---
Avera	---	4.1	0.28	4.1	4.1	2.2	1.1	400.9	5.0	4.7	---
SD(N)	---	0.0	0.00	0.3	0.3	0.1	0.1	77.7	1.2	0.6	---
CV%	---	0.0	1.53	6.9	6.9	3.8	12.9	19.4	24.0	12.3	---

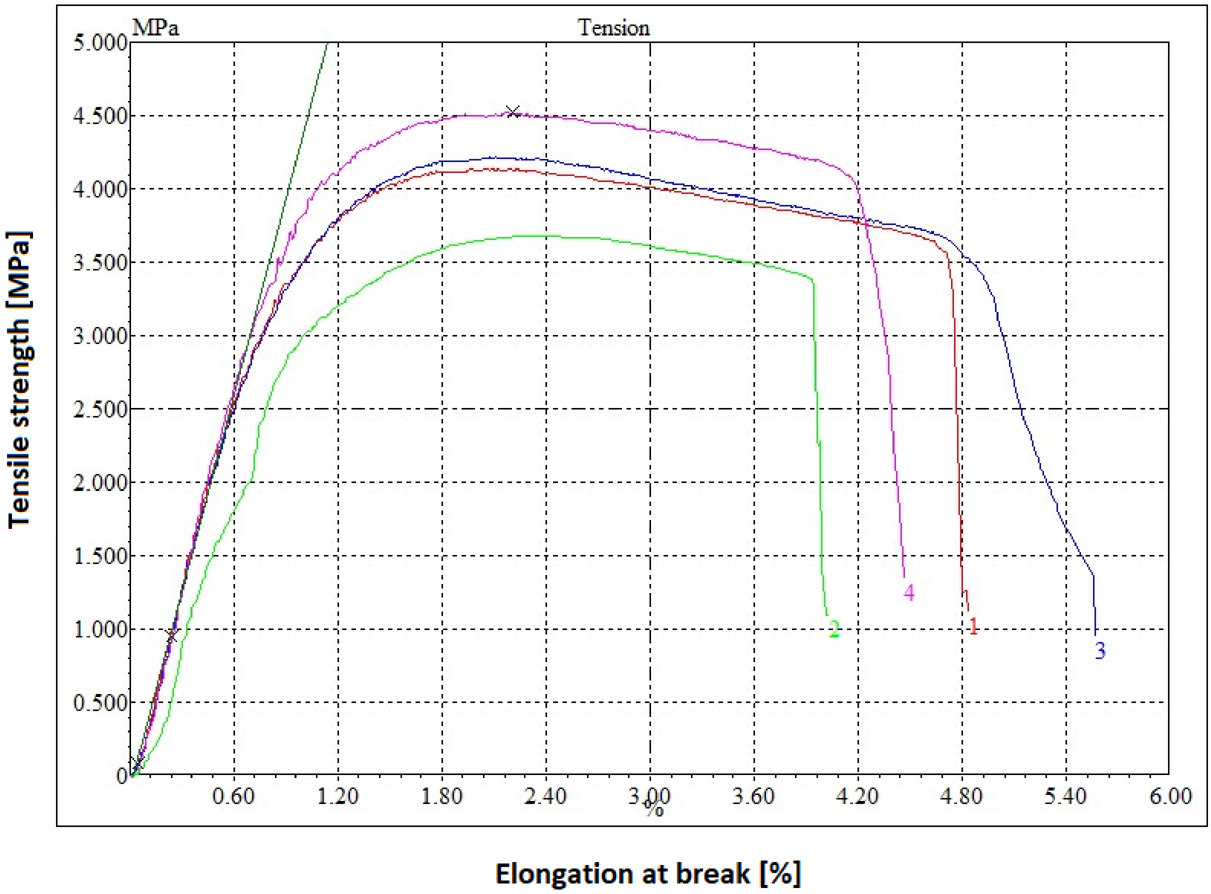

EVA 28150 + W.F. (60%)

Speed: 100.000 mm/min

Extrusion Grinev (Thickness: 1.0 mm) ; +21.8°C, 7.7%HR Test Data: 13-12-2017 Metod

Test No.	Material	Width mm	Thick ness mm	Max. Load MPa	Yeild point MPa	Max. Load %	Break MPa	Elastic modulus MPa	Input Grip %	Grip traversa %	Comentary °
1	EVA 28150 + W.F. (60%)	4.1	0.48	2.9	2.9	1.9	0.9	316.5	5.0	3.9	NO
2	EVA 28150 + W.F. (60%)	4.1	0.45	2.4	2.4	1.8	0.7	277.1	5.0	3.9	OK
3	EVA 28150 + W.F. (60%)	4.1	0.46	2.9	2.9	1.9	0.8	341.5	5.0	4.8	OK
4	EVA 28150 + W.F. (60%)	4.1	0.47	2.6	2.6	2.4	0.7	235.3	3.3	3.8	NO
5	EVA 28150 + W.F. (60%)	4.1	0.49	2.9	2.9	1.7	0.8	252.1	3.3	3.6	NO
---	---	---	---	---	---	---	---	---	---	---	---
Avera	---	4.1	0.47	2.7	2.7	1.9	0.8	284.5	4.3	4.0	---
SD(N)	---	0.0	0.01	0.2	0.2	0.2	0.1	39.5	0.8	0.4	---
CV%	---	0.0	3.01	7.5	7.5	12.5	9.6	13.9	19.3	10.4	---

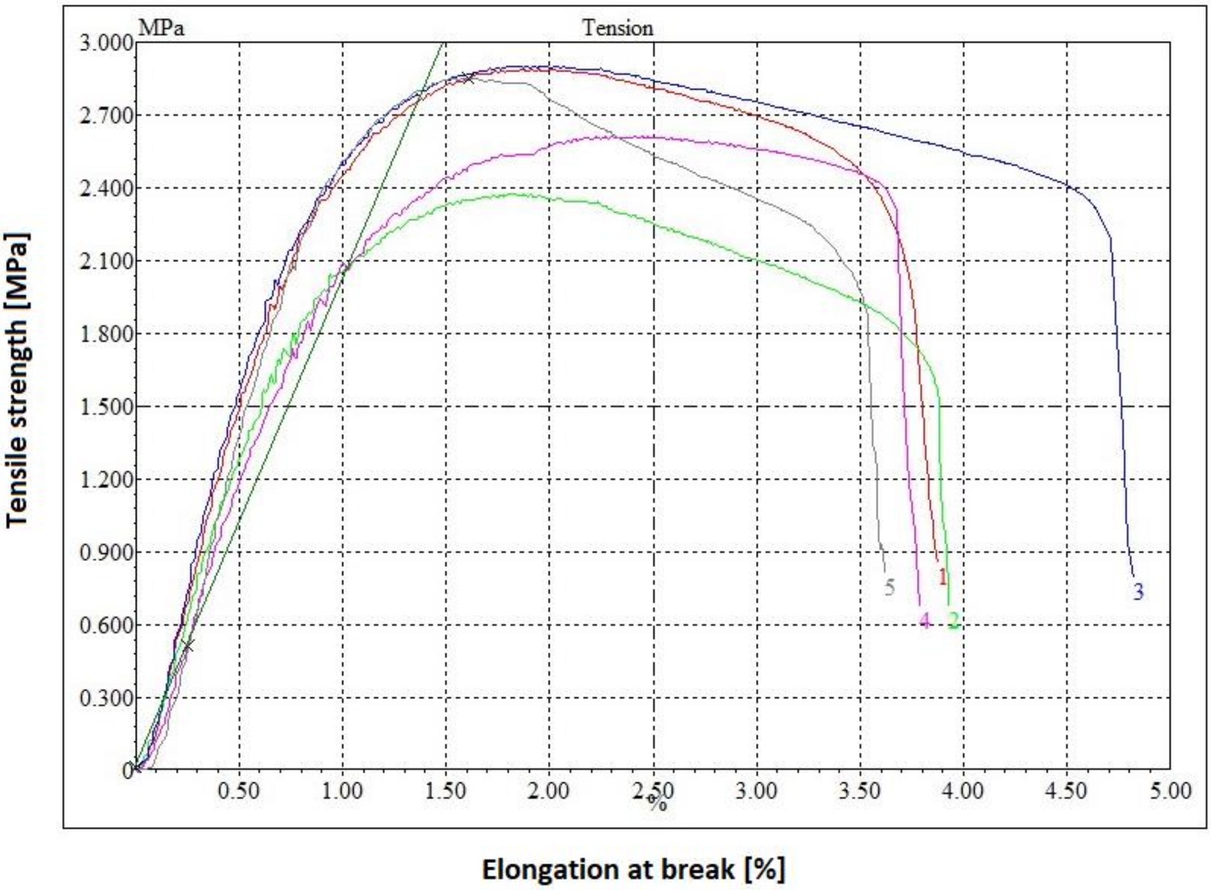

EVA 28025 + W.F. (60%)

Speed: 100.000 mm/min

Extrusion Grinev (Thickness: 1.0 mm) ; +21.8°C, 7.7%HR Test Data: 13-12-2017 Metod

Test No.	Material	Width mm	Thick ness mm	Max. Load MPa	Yeild point MPa	Max. Load %	Break MPa	Elastic modulus MPa	Input Grip %	Grip traversa %	Comentary °
1	EVA 28025 + W.F. (60%)	4.1	0.38	2.8	2.8	2.4	0.8	342.9	8.3	7.9	OK
2	EVA 28025 + W.F. (60%)	4.1	0.41	2.6	2.6	2.1	0.8	308.2	8.3	9.1	OK
3	EVA 28025 + W.F. (60%)	4.1	0.40	3.0	3.0	2.5	0.9	382.4	8.3	9.1	OK
---	---	---	---	---	---	---	---	---	---	---	---
---	---	---	---	---	---	---	---	---	---	---	---
---	---	---	---	---	---	---	---	---	---	---	---
Avera	---	4.1	0.40	2.8	2.8	2.3	0.8	344.5	8.3	8.7	---
SD(N)	---	0.0	0.01	0.2	0.2	0.2	0.0	30.3	0.0	0.6	---
CV%	---	0.0	3.14	5.8	5.8	7.3	5.7	8.8	0.0	6.5	---

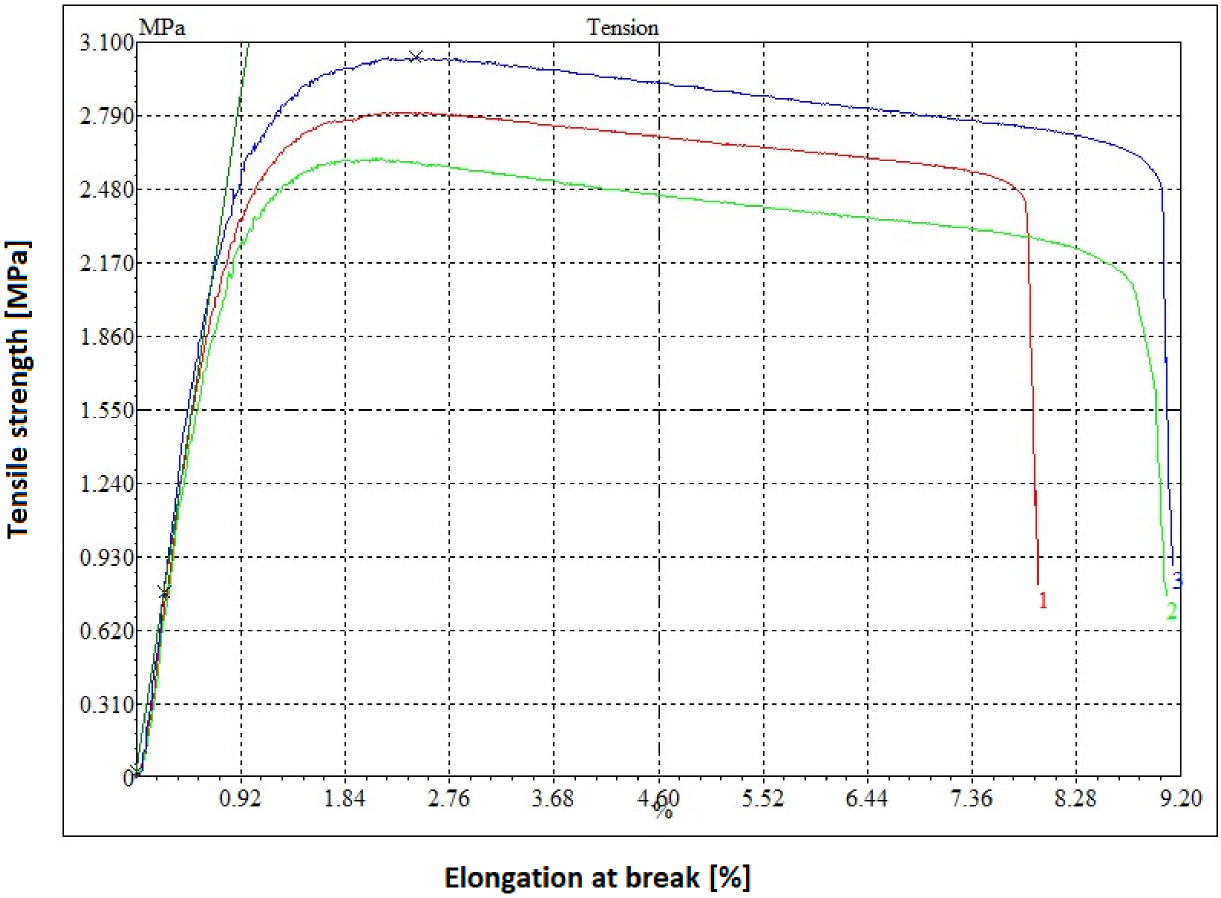

EVA 28005 + W.F. (60%)

Speed: 100.000 mm/min

Extrusion Grinev (Thickness: 1.0 mm) ; +21.8°C, 7.7%HR Test Data: 13-12-2017

Metod

Test No.	Material	Width mm	Thick ness mm	Max. Load MPa	Yeild point MPa	Max. Load %	Break MPa	Elastic modulus MPa	Input Grip %	Grip traversa %	Comentary °
1	EVA 28005 + W.F. (60%)	4.1	0.41	3.5	3.5	1.7	1.0	371.5	5.0	7.3	NO
2	EVA 28005 + W.F. (60%)	4.1	0.38	3.4	3.4	1.9	1.0	363.2	8.3	8.6	OK
3	EVA 28005 + W.F. (60%)	4.1	0.40	3.4	3.4	1.8	0.9	246.1	8.3	9.0	OK
---	---	---	---	---	---	---	---	---	---	---	---
---	---	---	---	---	---	---	---	---	---	---	---
---	---	---	---	---	---	---	---	---	---	---	---
Avera	---	4.1	0.40	3.4	3.4	1.8	1.0	326.9	7.2	8.3	---
SD(N)	---	0.0	0.01	0.0	0.0	0.1	0.0	57.3	1.6	0.7	---
CV%	---	0.0	3.14	1.4	1.4	4.5	4.9	17.5	21.6	8.7	---

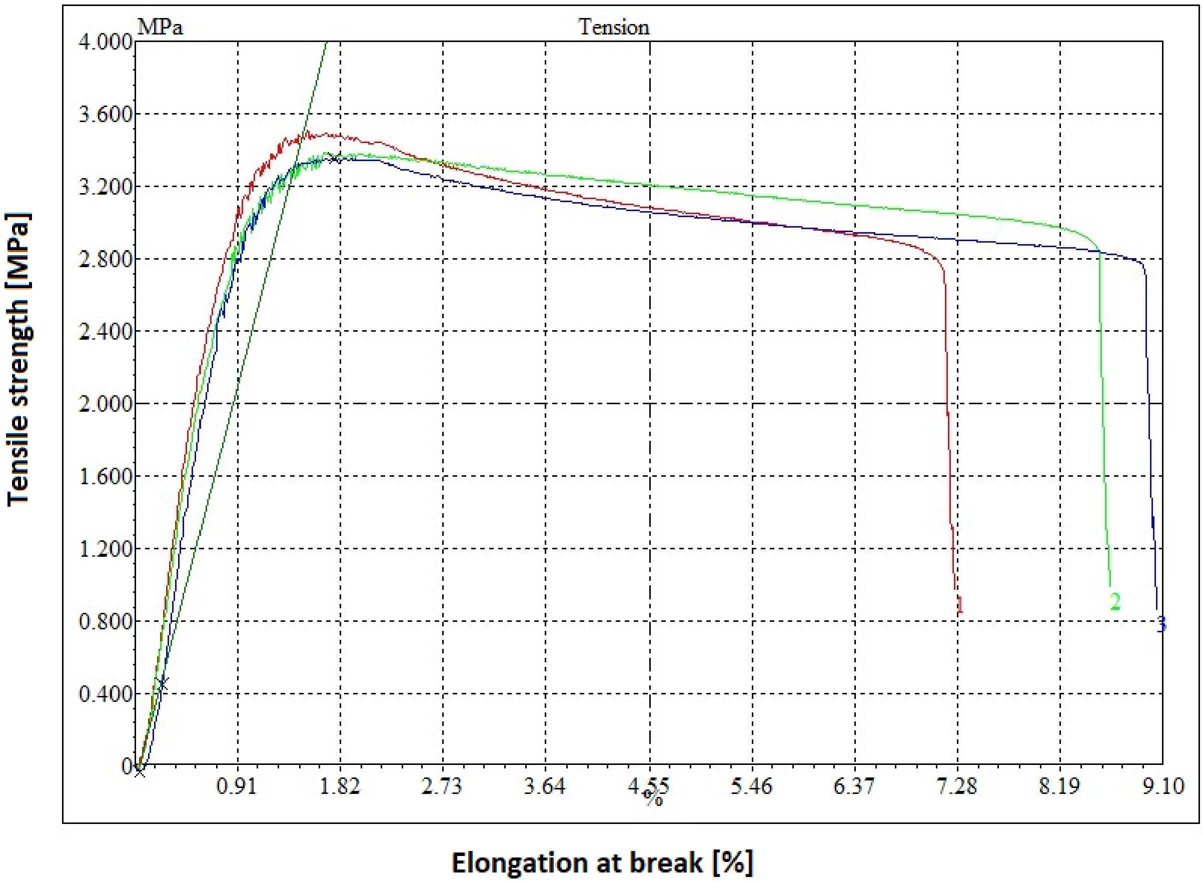

EVA 19150 + W.F. (60%)

Speed: 100.000 mm/min

Extrusion Grinev (Thickness: 1.0 mm) ; +21.8°C, 7.7%HR Test Data: 13-12-2017 Metod

Test No.	Material	Width mm	Thick ness mm	Max. Load MPa	Yeild point MPa	Max. Load %	Break MPa	Elastic modulus MPa	Input Grip %	Grip traversa %	Comentary °
1	EVA 19150 + W.F. (60%)	4.1	0.41	2.6	2.6	1.8	0.7	307.7	1.7	2.5	OK
2	EVA 19150 + W.F. (60%)	4.1	0.42	3.0	3.0	2.0	0.8	395.7	1.7	2.7	OK
3	EVA 19150 + W.F. (60%)	4.1	0.41	2.8	2.8	-	0.7	392.1	1.7	1.6	OK
4	EVA 19150 + W.F. (60%)	4.1	0.44	3.0	3.0	2.1	0.9	336.9	1.7	2.7	NO
5	EVA 19150 + W.F. (60%)	4.1	0.38	2.7	2.7	-	0.8	272.7	1.7	1.7	NO
---	---	---	---	---	---	---	---	---	---	---	---
Avera	---	4.1	0.41	2.8	2.8	2.0	0.8	341.0	1.7	2.2	---
SD(N)	---	0.0	0.02	0.2	0.2	0.1	0.1	47.7	0.0	0.5	---
CV%	---	0.0	4.71	5.7	5.7	6.3	9.6	14.0	0.0	21.8	---

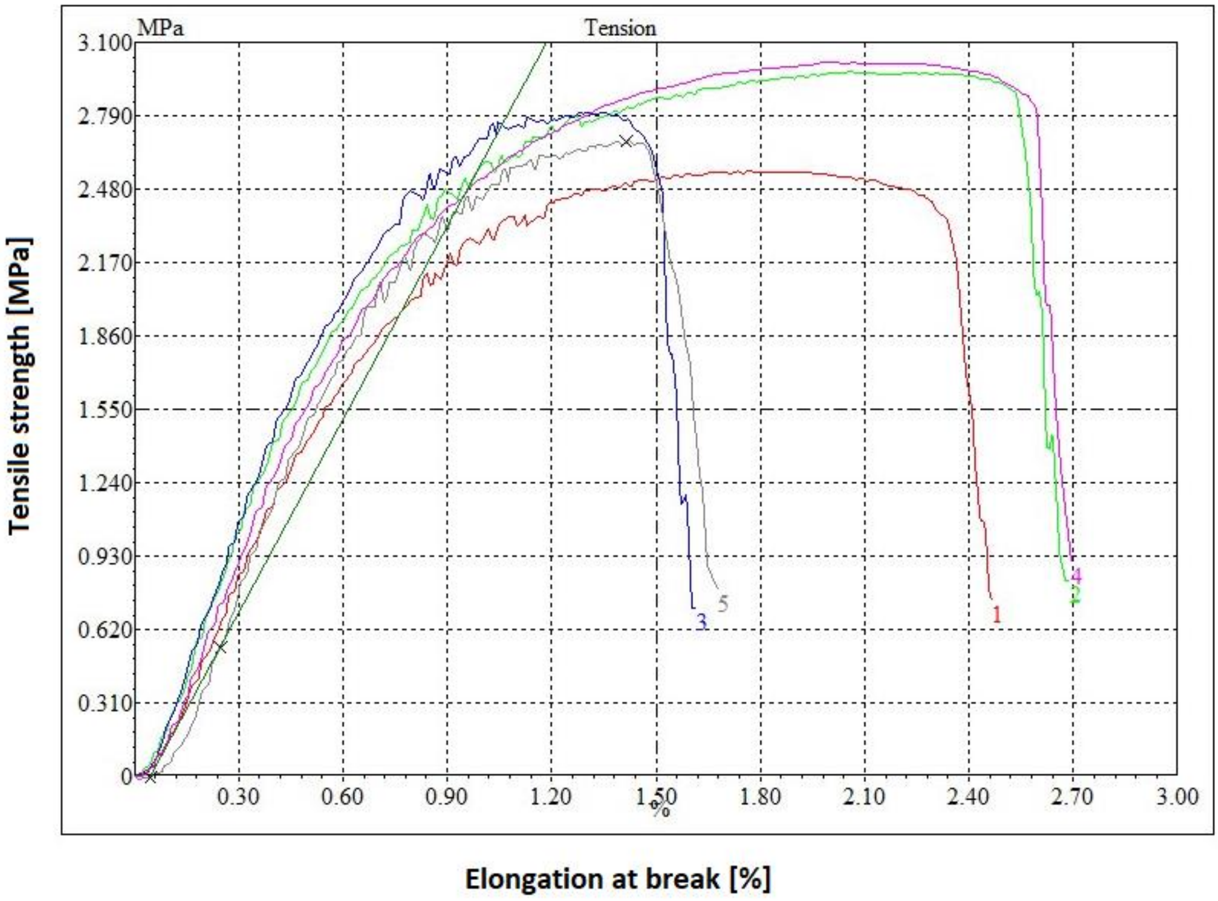

EVA 15006 + W.F. (60%)

Speed: 100.000 mm/min

Extrusion Grinev (Thickness: 1.0 mm) ; +21.8°C, 7.7%HR Test Data: 13-12-2017 Metod

Test No.	Material	Width mm	Thick ness mm	Max. Load MPa	Yeild point MPa	Max. Load %	Break MPa	Elastic modulus MPa	Input Grip %	Grip traversa %	Comentary º
1	EVA 15006 + W.F. (60%)	4.1	0.49	3.9	3.9	2.1	1.1	391.1	3.3	3.5	NO
2	EVA 15006 + W.F. (60%)	4.1	0.46	5.0	5.0	1.7	1.5	530.5	1.7	2.2	NO
3	EVA 15006 + W.F. (60%)	4.1	0.45	5.2	5.2	1.7	1.5	529.9	1.7	2.1	OK
---	---	---	---	---	---	---	---	---	---	---	---
---	---	---	---	---	---	---	---	---	---	---	---
---	---	---	---	---	---	---	---	---	---	---	---
Avera	---	4.1	0.47	4.7	4.7	1.8	1.4	483.8	2.2	2.6	---
SD(N)	---	0.0	0.02	0.6	0.6	0.2	0.2	65.6	0.8	0.6	---
CV%	---	0.0	3.64	12.2	12.2	10.3	13.8	13.6	33.8	24.5	---

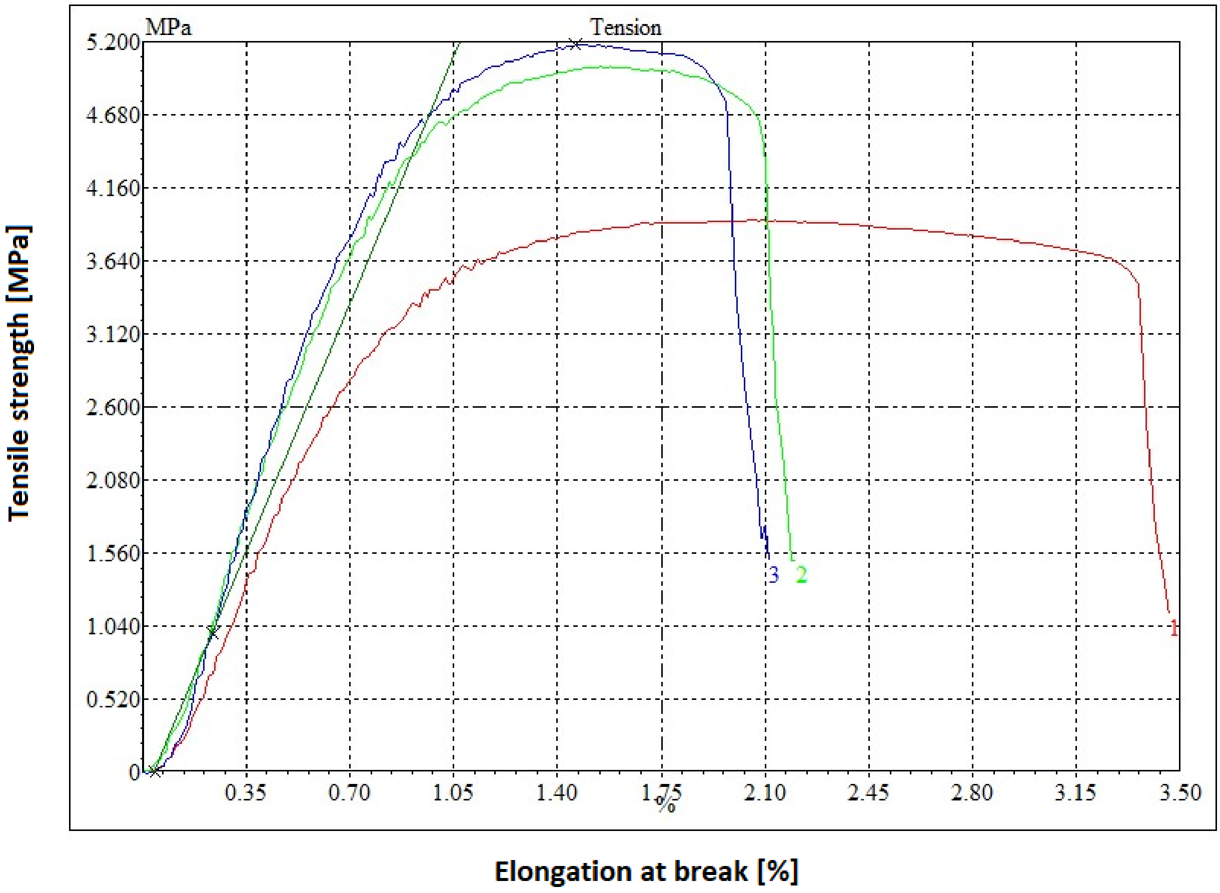

EVA 28150 + W.F (70%)

Speed: 100.000 mm/min

Extrusion Grinev (Thickness: 1.0 mm) ; +21.8°C, 7.7%HR Test Data: 14-12-2017

Metod

Test No.	Material	Width mm	Thick ness mm	Max. Load MPa	Yeild point MPa	Max. Load %	Break MPa	Elastic modulus MPa	Input Grip %	Grip traversa %	Comentary °
1	EVA 28150 + W.F (70%)	4.1	0.59	2.1	2.1	3.1	0.6	201.2	5.0	4.5	OK
2	EVA 28150 + W.F (70%)	4.1	0.60	2.0	2.0	2.8	0.4	212.6	5.0	4.0	OK
3	EVA 28150 + W.F (70%)	4.1	0.57	2.4	2.4	3.1	0.7	229.4	5.0	4.2	NO
---	---	---	---	---	---	---	---	---	---	---	---
---	---	---	---	---	---	---	---	---	---	---	---
---	---	---	---	---	---	---	---	---	---	---	---
Avera	---	4.1	0.59	2.2	2.2	3.0	0.6	214.4	5.0	4.2	---
SD(N)	---	0.0	0.01	0.2	0.2	0.1	0.1	11.6	0.0	0.2	---
CV%	---	0.0	2.13	7.8	7.8	4.7	22.0	5.4	0.0	4.9	---

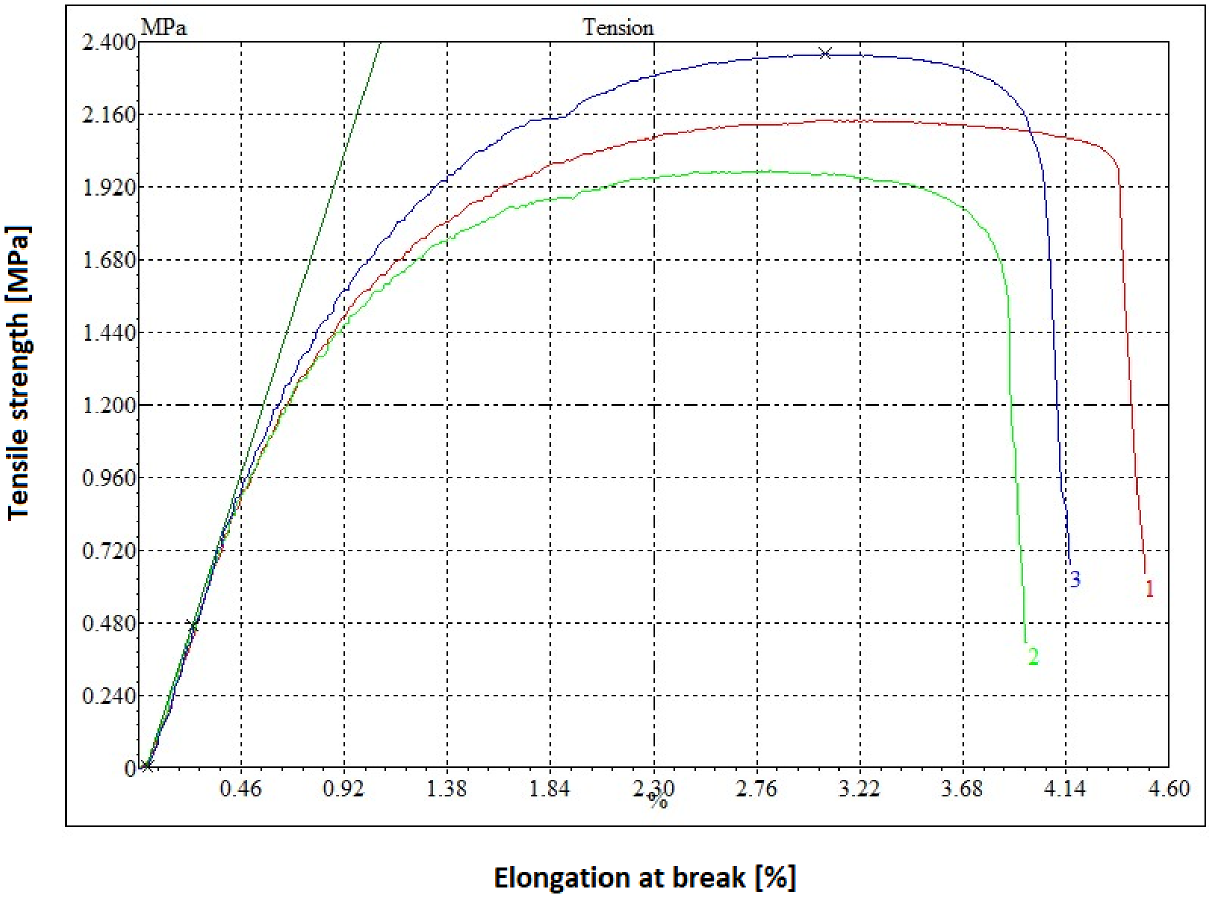

EVA 28025 + W.F (70%)

Speed: 100.000 mm/min

Extrusion Grinev (Thickness: 1.0 mm) ; +21.8°C, 7.7%HR Test Data: 14-12-2017

Metod

Test No.	Material	Width mm	Thick ness mm	Max. Load MPa	Yeild point MPa	Max. Load %	Break MPa	Elastic modulus MPa	Input Grip %	Grip traversa %	Comentary °
1	EVA 28025 + W.F (70%)	4.1	0.48	2.6	2.6	3.1	0.8	229.0	6.7	6.2	OK
2	EVA 28025 + W.F (70%)	4.1	0.50	2.5	2.5	3.8	0.7	191.1	11.7	10.7	OK
3	EVA 28025 + W.F (70%)	4.1	0.49	2.4	2.4	3.7	0.6	173.7	10.0	8.9	OK
4	EVA 28025 + W.F (70%)	4.1	0.50	2.5	2.5	3.1	0.7	177.9	5.0	4.1	OK
---	---	---	---	---	---	---	---	---	---	---	---
---	---	---	---	---	---	---	---	---	---	---	---
Avera	---	4.1	0.49	2.5	2.5	3.4	0.7	192.9	8.3	7.5	---
SD(N)	---	0.0	0.01	0.1	0.1	0.3	0.1	21.8	2.6	2.5	---
CV%	---	0.0	1.68	2.8	2.8	9.5	10.1	11.3	31.6	33.7	---

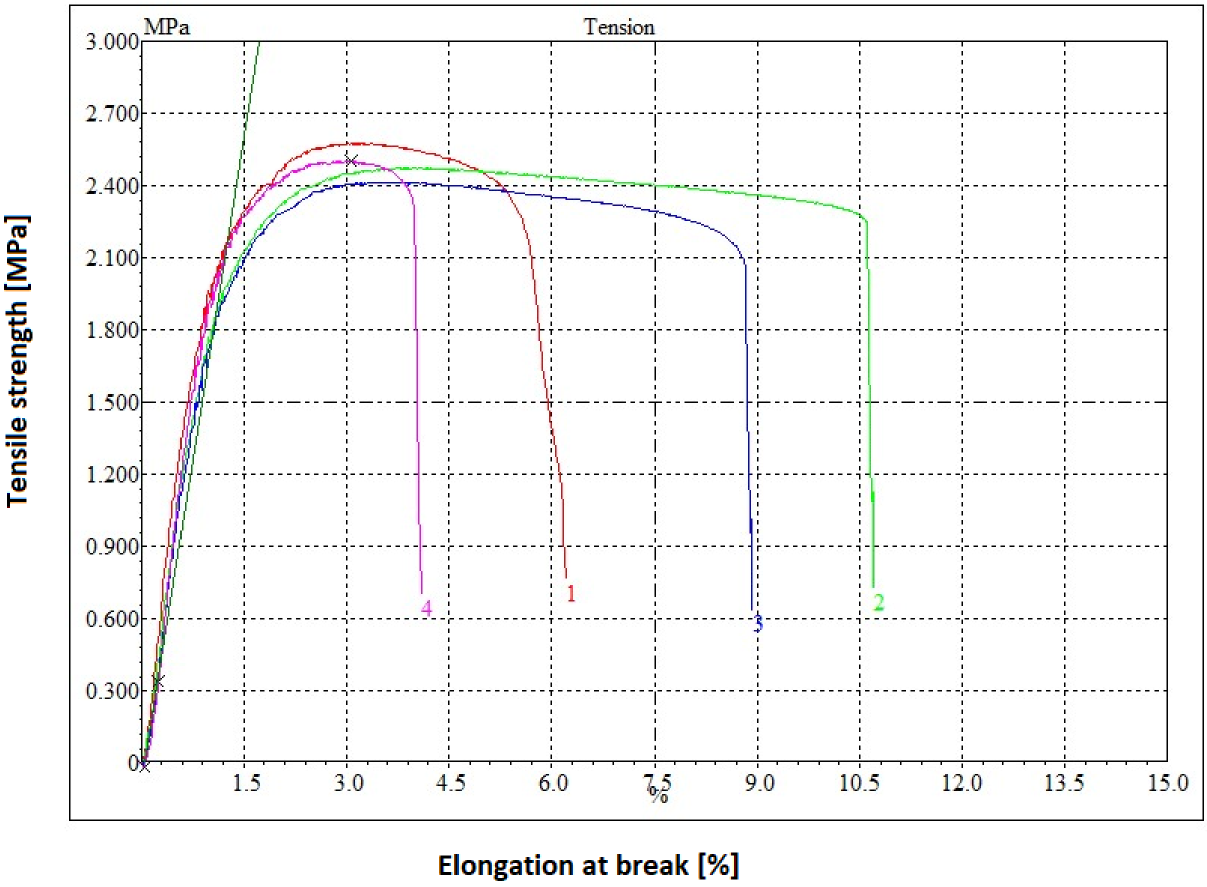

EVA 28005 + W.F (70%)

Speed: 100.000 mm/min

Extrusion Grinev (Thickness: 1.0 mm) ; +21.8°C, 7.7%HR Test Data: 14-12-2017 Metod

Test No.	Material	Width mm	Thick ness mm	Max. Load MPa	Yeild point MPa	Max. Load %	Break MPa	Elastic modulus MPa	Input Grip %	Grip traversa %	Comentary °
1	EVA 28005 + W.F (70%)	4.1	0.56	3.3	3.3	3.2	0.9	146.1	5.0	4.3	OK
2	EVA 28005 + W.F (70%)	4.1	0.60	3.4	3.4	2.7	0.9	154.2	5.0	3.4	OK
3	EVA 28005 + W.F (70%)	4.1	0.57	3.2	3.2	2.6	0.9	272.6	3.3	3.5	NO
---	---	---	---	---	---	---	---	---	---	---	---
---	---	---	---	---	---	---	---	---	---	---	---
---	---	---	---	---	---	---	---	---	---	---	---
Avera	---	4.1	0.58	3.3	3.3	2.8	0.9	191.0	4.4	3.7	---
SD(N)	---	0.0	0.02	0.1	0.1	0.3	0.0	57.8	0.8	0.4	---
CV%	---	0.0	2.95	2.5	2.5	9.3	0.0	30.3	18.1	10.8	---

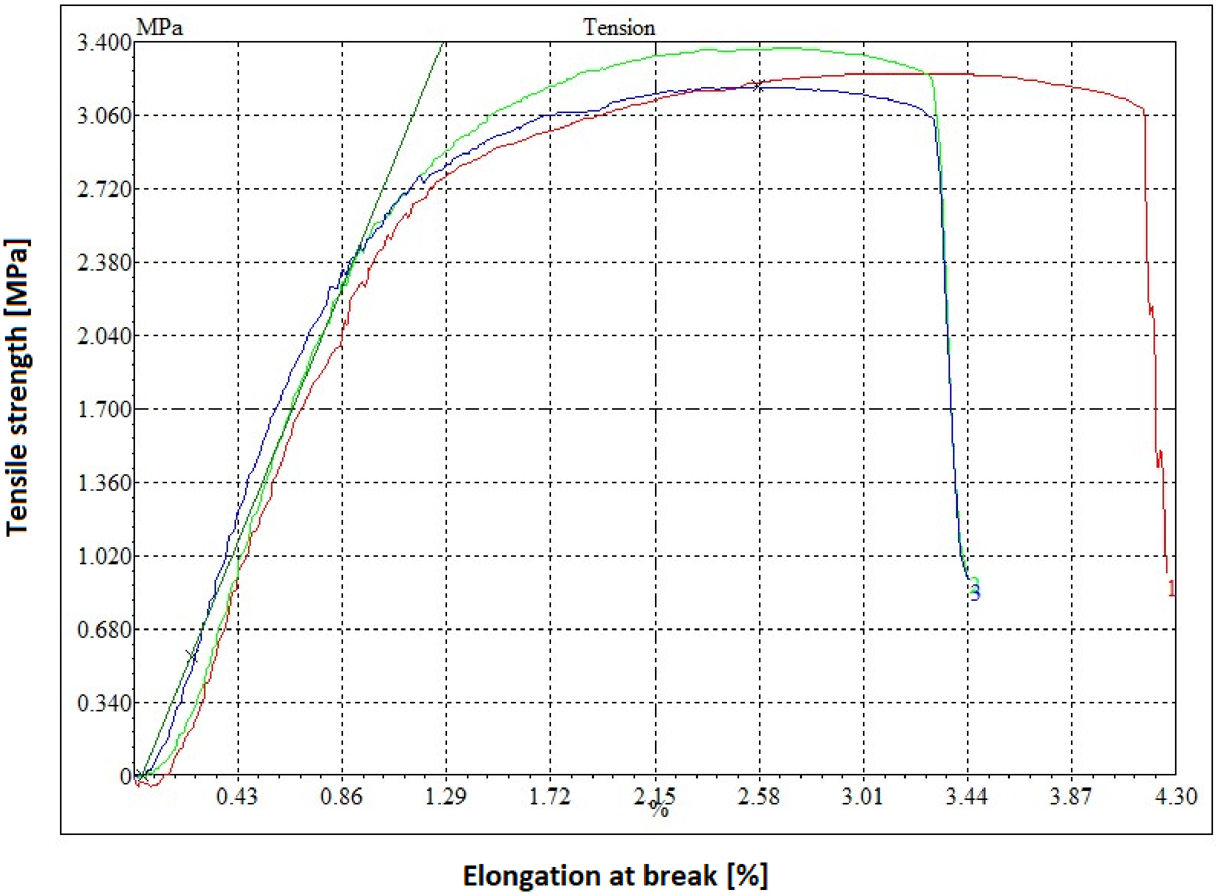

EVA 19150+ W.F (70%)

Speed: 100.000 mm/min

Extrusion Grinev (Thickness: 1.0 mm) ; +21.8°C, 7.7%HR Test Data: 14-12-2017

Metod

Test No.	Material	Width	Thick ness	Max. Load	Yeild point	Max. Load	Break	Elastic modulus	Input Grip	Grip traversa	Comentary
		mm	mm	MPa	MPa	%	MPa	MPa	%	%	°
1	EVA 19150+ W.F (70%)	4.1	0.52	2.3	2.3	-	0.6	352.3	1.7	1.4	OK
2	EVA 19150+ W.F (70%)	4.1	0.58	1.9	1.9	-	0.6	312.0	1.7	1.1	OK
3	EVA 19150+ W.F (70%)	4.1	0.53	2.1	2.1	-	0.5	343.2	1.7	1.5	OK
---	---	---	---	---	---	---	---	---	---	---	---
---	---	---	---	---	---	---	---	---	---	---	---
---	---	---	---	---	---	---	---	---	---	---	---
Avera	---	4.1	0.54	2.1	2.1	0.0	0.6	335.8	1.7	1.3	---
SD(N)	---	0.0	0.03	0.2	0.2	0.0	0.0	17.3	0.0	0.2	---
CV%	---	0.0	4.83	7.8	7.8	0.0	8.3	5.1	0.0	12.7	---

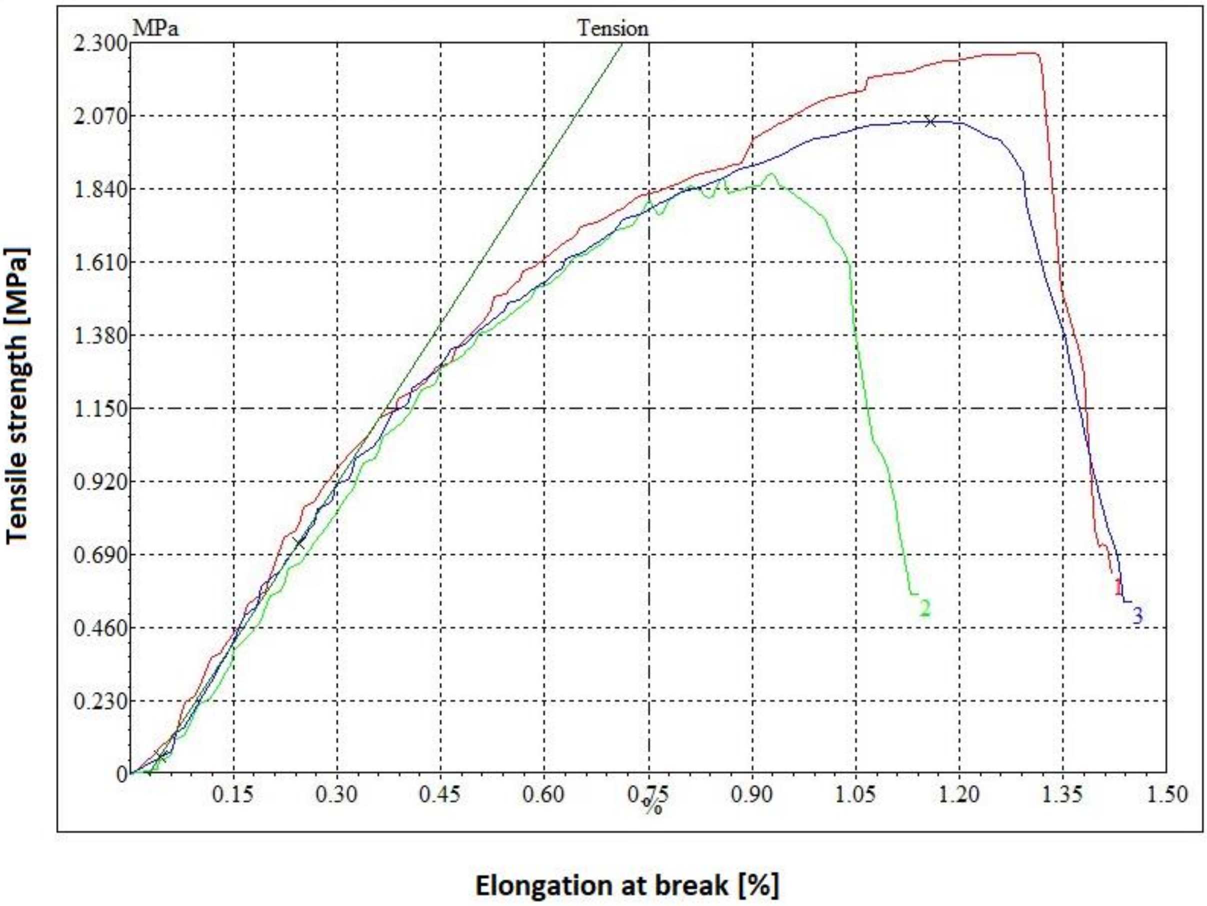

EVA 15006+ W.F (70%)

Speed: 100.000 mm/min

Extrusion Grinev (Thickness: 1.0 mm) ; +21.8°C, 7.7%HR Test Data: 14-12-2017

Metod

Test No.	Material	Width mm	Thickness mm	Max. Load MPa	Yeild point MPa	Max. Load %	Break MPa	Elastic modulus MPa	Input Grip %	Grip traversa %	Comentary °
1	EVA 15006+ W.F (70%)	4.1	0.59	2.7	2.7	-	0.7	177.0	1.7	1.6	OK
2	EVA 15006+ W.F (70%)	4.1	0.59	2.8	2.8	-	0.8	199.3	1.7	1.5	NO
3	EVA 15006+ W.F (70%)	4.1	0.58	3.0	3.0	1.7	0.9	204.4	1.7	1.9	OK
---	---	---	---	---	---	---	---	---	---	---	---
---	---	---	---	---	---	---	---	---	---	---	---
---	---	---	---	---	---	---	---	---	---	---	---
Avera	---	4.1	0.59	2.8	2.8	1.7	0.8	193.6	1.7	1.7	---
SD(N)	---	0.0	0.00	0.1	0.1	0.0	0.1	11.9	0.0	0.2	---
CV%	---	0.0	0.80	4.4	4.4	0.0	10.2	6.1	0.0	10.2	---

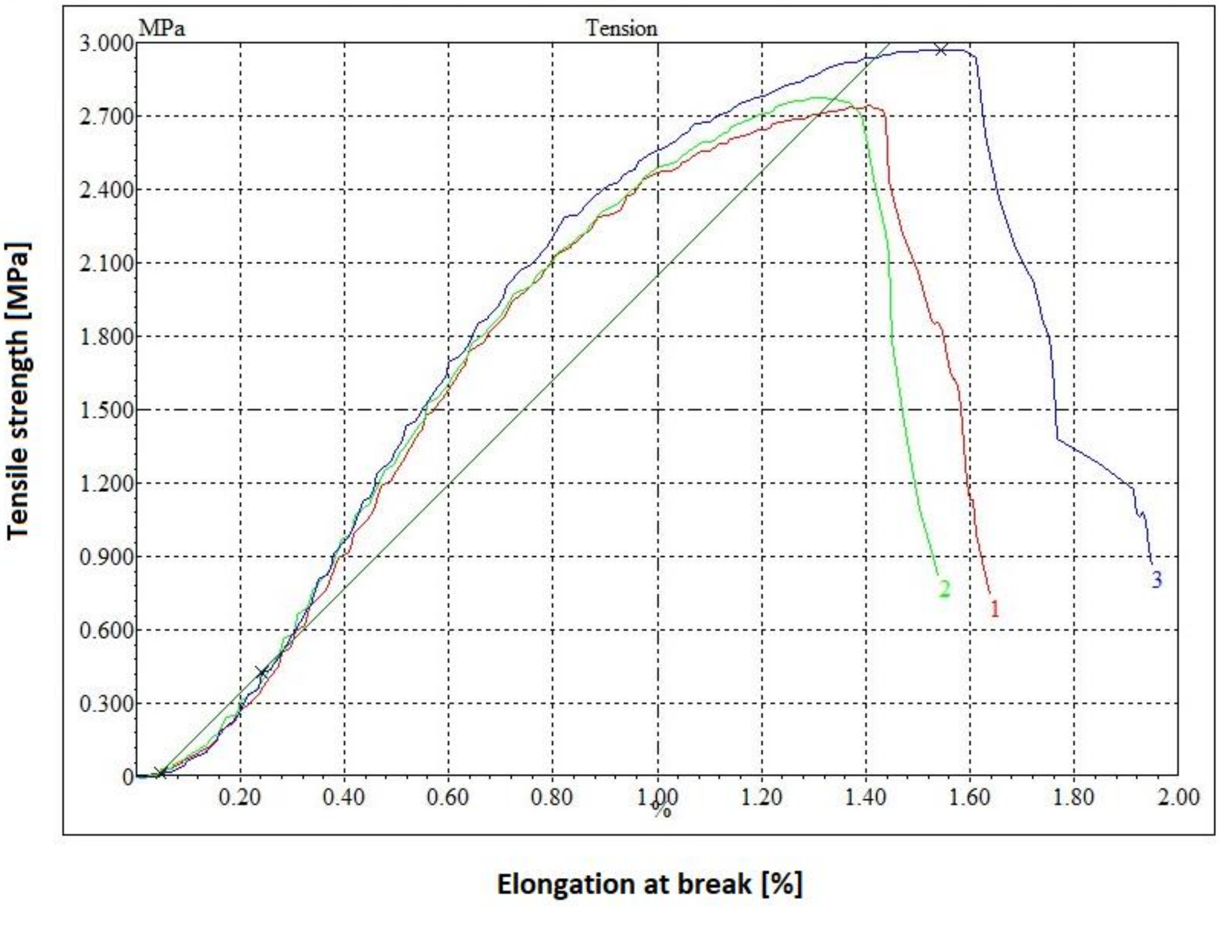

Supplement: Supplementary file 1 [file polymers-15-02639-s001.zip › polymers-2402232-supplementary.pdf]
